# Function of cofactor Akirin2 in the regulation of gene expression in model human Caucasian neutrophil-like HL60 cells

**DOI:** 10.1042/BSR20211120

**Published:** 2021-07-22

**Authors:** Sara Artigas-Jerónimo, Margarita Villar, Agustín Estrada-Peña, Adrián Velázquez-Campoy, Pilar Alberdi, José de la Fuente

**Affiliations:** 1 SaBio, Instituto de Investigación en Recursos Cinegéticos IREC-CSIC-UCLM-JCCM, Ronda de Toledo s/n, Ciudad Real 13005, Spain; 2 Biochemistry Section, Faculty of Science and Chemical Technologies, and Regional Centre for Biomedical Research (CRIB), University of Castilla-La Mancha, Ciudad Real 13071, Spain; 3 Department of Animal Pathology, Faculty of Veterinary Medicine, Zaragoza 50013, Spain; 4 Institute of Biocomputation and Physics of Complex Systems (BIFI), Joint Units IQFR-CSIC-BIFI, and GBsC-CSIC-BIFI, Universidad de Zaragoza, Zaragoza 50018, Spain; 5 Departamento de Bioquímica y Biología Molecular y Celular, Universidad de Zaragoza 50009, Zaragoza, Spain; 6 Instituto de Investigación Sanitaria Aragón (IIS Aragón), Zaragoza 50009, Spain; 7 Centro de Investigación Biomédica en Red en el Área Temática de Enfermedades Hepáticas y Digestivas (CIBERehd), Madrid 28029, Spain; 8 Fundación ARAID, Gobierno de Aragón, Zaragoza 50009, Spain; 9 Department of Veterinary Pathobiology, Center for Veterinary Health Sciences, Oklahoma State University, Stillwater, OK 74078, U.S.A.

**Keywords:** Akirin, epigenetics, gene regulatory networks, histones, interactome, Subolesin

## Abstract

The Akirin family of transcription cofactors are involved throughout the metazoan in the regulation of different biological processes (BPs) such as immunity, interdigital regression, muscle and neural development. Akirin do not have catalytic or DNA-binding capability and exert its regulatory function primarily through interacting proteins such as transcription factors, chromatin remodelers, and RNA-associated proteins. In the present study, we focused on the human Akirin2 regulome and interactome in neutrophil-like model human Caucasian promyelocytic leukemia HL60 cells. Our hypothesis is that metazoan evolved to have Akirin2 functional complements and different Akirin2-mediated mechanisms for the regulation of gene expression. To address this hypothesis, experiments were conducted using transcriptomics, proteomics and systems biology approaches in *akirin2* knockdown and wildtype (WT) HL60 cells to characterize Akirin2 gene/protein targets, functional complements and to provide evidence of different mechanisms that may be involved in Akirin2-mediated regulation of gene expression. The results revealed Akirin2 gene/protein targets in multiple BPs with higher representation of immunity and identified immune response genes as candidate Akirin2 functional complements. In addition to linking chromatin remodelers with transcriptional activation, Akirin2 also interacts with histone H3.1 for regulation of gene expression.

## Introduction

The Akirin (also known as Subolesin (SUB) in ticks) family constitute a model for the study of host–pathogen molecular interactions and functional biology due to their conserved function throughout the metazoan in the regulation of different biological processes (BPs) such as immunity, interdigital regression, muscle and neural development [1–10]. Akirin are considered transcription cofactors without catalytic or DNA-binding capability involved in the regulation of signaling pathways such as immune deficiency (IMD), tumor necrosis factor (TNF)/Toll-like receptor (TLR)-nuclear factor κ-light-chain-enhancer of activated B cells (NF-κB) (TNF/TLR) and ATP-dependent SWI/SNF-like Brg1/Brm-associated factor (BAF) chromatin remodeling complexes [1,5,11–14]. It has been shown that Akirin proteins exert its regulatory function primarily through interaction with other proteins (Akirin-interacting proteins, IPs) such as transcription factors, chromatin remodelers, and RNA-associated proteins [2,7,9,13,14–21]. In particular, human Akirin2 (Akirin2), the functional homolog of *Drosophila melanogaster* Akirin and tick SUB, has been shown to regulate various BPs in a biological context, cell type and stimulus-dependent manner, by acting as a link between NF-κB and chromatin remodeling complexes (remodelers) for transcriptional regulation [14,22].

The epigenetic regulation is defined as processes mediating phenotype changes without genotype changes [23]. The processes of epigenetic regulation include DNA modifications, non-coding RNAs, histone post-translational modifications and chromatin remodeling [24]. Chromatin packages the DNA into chromosomal higher order structures, which condenses and organizes the genome. Although chromatin can obstruct several regulatory elements, it also allows chromatin components to actively participate in the regulation of transcription, chromosome segregation, DNA replication and DNA repair [22]. Consequently, cells have evolved a set of specialized chromatin remodelers to alter nucleosome composition in chromosomal regions [22]. Chromatin remodeling controls DNA accessibility for the binding of transcription regulators that play a key role in the regulation of various BPs such as immune response and development [25]. This process is regulated at different levels such as NF-κB-mediated regulation after recruitment by Akirin2 of the SWI/SNF complex to the transcription factor IκBζ [3].

The regulome (transcription factors/cofactors–target genes interactions) and interactome (protein–protein physical and functional interactions) play a critical role in the regulation of cell BPs [26,27]). Therefore, the application of regulomics and interactomics to key transcriptional regulators such as Akirin is important to better understand their role in host–pathogen interactions, cell biology and regulation of gene expression.

In the present study, we focused on the Akirin2 regulome and interactome in model human Caucasian promyelocytic leukemia HL60 cells to advance the knowledge of the function of this highly conserved regulatory cofactor. Human neutrophils play a central role in innate immunity, and HL60 cells are used as a neutrophil-like model [28] with reported expression of *akirin* in uninfected and *Anaplasma*-infected cells [29]. The key function of Akirin2 in cell interactome, regulome and epigenetic gene regulation [14,22] translated into the question of how Akirin2 regulate gene expression in human HL60 cells? This question raised the hypothesis that metazoan evolved to have Akirin2 functional complements (proteins that exercise their function in BPs in which Akirin2 is involved to complement its function in case of genetic knockdown) and different mechanisms for Akirin2-mediated regulation of gene expression. To address this hypothesis, experiments were conducted using transcriptomics, proteomics and systems biology approaches in *akirin2* knockdown (KO) and wildtype (WT) human HL60 cells to characterize Akirin2 gene/protein targets, functional complements and to provide evidence of different mechanisms that may be involved in Akirin2-mediated regulation of gene expression. The results revealed Akirin2 gene/protein targets in multiple BPs with higher representation of immunity, identified immune response genes as candidate Akirin2 functional complements and showed that Akirin2 is not only a link with chromatin remodelers, but also directly interacts with histone H3.1, thus providing new information on the regulome and interactome of this transcription cofactor.

## Materials and methods

### Experimental design and rationale

**Figure 1:**
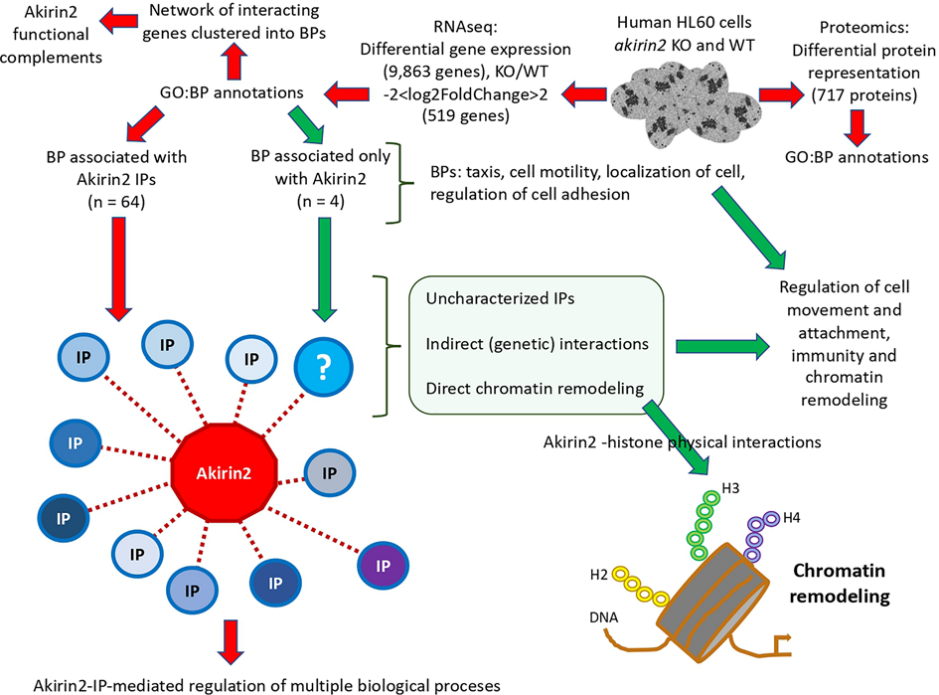
Experimental design and rationale The effect of *akirin2* KO was evaluated on the transcriptome and proteome of human HL60 cells. Results of differential gene expression and protein representation in *akirin2* KO and WT cells and analysis of GO annotations for BPs were used to characterize Akirin2 gene/protein targets and to identify Akirin2 functional complements. The BPs that are not related to known Akirin2 IPs and thus associated only with Akirin2 were identified and selected for further analysis (green arrows). Genes in these BPs may be regulated by Akirin2 through unknown IPs, indirect (genetic) interactions or direct chromatin remodeling. To address the possibility of direct chromatin remodeling, Akirin2–histone physical interactions were then investigated.

The experimental design and rationale are described in Figure 1. In the present study, we used the model HL60 cells (ATCC CCL-240) [28]. Transcriptomics and proteomics analyses of differential gene expression and protein representation in response to *akirin2* KO when compared with WT human HL60 cells was used to characterize *akirin2* gene/Akirin 2 protein targets (Supplementary Data S1–S3). Genes showing a KO/WT −2 < log2FoldChange > 2 were selected for further analysis. Gene ontology (GO) annotations and analyses for BPs were conducted for differentially regulated genes and proteins to identify those that are highly linked to Akirin2 (Supplementary Data S1 and S3). Then, a graph theory algorithm was used to characterize the network of interacting genes clustered into BPs to identify Akirin2 functional complements that show high up-regulation and centrality index or relative importance in response to *akirin2* KO (Supplementary Data S4). The BPs that are not related to known Akirin2 IPs were identified as BPs that may be regulated by Akirin2 through unknown IPs, indirect (genetic) interactions or direct Akirin2–histone interactions (Supplementary Data S5). Finally, the Akirin2–histone physical interactions were characterized using four experimental approaches: (i) discovery of putative Akirin2–histone interactions by recombinant Akirin2 (recAkirin2) binding to a histone peptide array (Supplementary Data S6), (ii) corroboration of recAkirin2–histone H3.1 interaction by *in vitro* protein pull-down and Western blot analysis, (iii) corroboration of recAkirin2–histone H3.1 interaction by isothermal titration calorimetry (ITC) and (iv) putative functional implication of Akirin2–histone H3.1 interaction in the regulation of gene expression.

### Human HL60 cells

Clustered Regularly Interspaced Short Palindromic Repeats (CRISPR-Cas9) *akirin2* KO (transfected with synthetic guide RNA/Cas9 ribonucleoprotein complex sgRNA/Cas9 RNP with sequence CAGAGUGGCUCCGCACGCCA targeting *akirin2* exons 6 and 7; Supplementary Data S2) and WT (transfected with pyogenes SpCas9 nuclease only to be used as the non-editing control) HL60 cells were provided by Synthego (Menlo Park, California, U.S.A.). In this way, CRISPR-edited *akirin2* KO cells were compared with WT cells created under the same conditions and transfected with SpCas9 only. Cells achieved an editing efficiency of 78% genotypic knockout determined by inference of CRISPR edits analysis (ICE; https://www.synthego.com/products/bioinformatics/crispr-analysis) and 75% protein knockout determined by flow cytometry. The cells were maintained in RPMI 1640 medium (Invitrogen, Carlsbad, CA, U.S.A.) supplemented with fetal bovine serum (FBS, Gibco Thermo Fisher Scientific, Waltham, MA, U.S.A.) at a concentration of 10% without antibiotics in a humidified incubator with 5% carbon dioxide at 37°C and used for RNA sequencing (RNAseq) without clonal expansion.

### RNAseq transcriptomics data acquisition and analysis in human HL60 cells

For RNAseq analysis of HL60 cells, we compared *akirin2* KO with WT cells as previously shown in CRISPR-Cas9 experiments (i.e. [30]) (Supplementary Data S2). RNA was isolated from WT and *akirin2* KO HL60 cells (three biological replicates with 8 × 10^6^ cells each) using the AllPrep DNA/RNA/Protein Mini Kit (Qiagen, Hilden, Germany). Purified RNA was used for RNAseq at the Genewiz’s service (Genewiz, South Plainfield, NJ, U.S.A.) (Supplementary Data S1). The Student’s *t* test (*P*<0.05) was used to perform two-sample comparisons between the averaged reads derived from each gene across the three replicate runs for each sample under comparison in order to identify genes that were significantly differentially regulated between WT and *akirin2* KO HL60 cells. Significantly regulated genes in response to *akirin2* KO with a −2 < log2 fold change > −2 were selected for further analyses. Functional GO:BP (level 3) annotations were done based on NCBI (https://www.ncbi.nlm.nih.gov/protein/) protein references for each of the selected genes using Blast2GO software (version 5.2; www.blast2go.com; accessed on 4 August 2020). For Akirin2 IPs included in BioGRID3.5.188 (https://thebiogrid.org/120430/table/homo-sapiens/akirin2.html?sort=evidence; accessed on 4 August 2020) and identified by Artigas-Jerónimo et al. [14], proteins ID were annotated based on NCBI references and GO:BP (level 3) annotations (Supplementary Data S5). Then, the BPs and differentially regulated genes associated with IPs or only with Akirin2 were identified (Supplementary Data S5). The annotations of the BPs associated only with Akirin2 and for GO:BP chromatin remodeling were obtained from QuickGO (https://www.ebi.ac.uk/QuickGO/; accessed on 4 August 2020) (Supplementary Data S5). Heatmaps were prepared using the Heatmapper (http://www.heatmapper.ca/expression/) with average linkage and Euclidean distance measurement method. Data were deposited at NCBI under study name ‘Effect of Akirin2 knockdown on the mRNA profile of human HL60 cells’ and GEO accession number GSE158885 (https://www.ncbi.nlm.nih.gov/geo/query/acc.cgi?acc=GSE158885).

### Gene expression analysis by qRT-PCR

The total RNA was extracted from WT and *akirin2* KO HL60 cells and used to characterize the mRNA levels of selected Akirin2 IPs by qRT-PCR using gene-specific oligonucleotide forward (F) and reverse (R) primers (*akirin2*, F: 5′-CGGAGCCACTCTGAAAAGGA-3′, R: 5′-GAGATACTTCTGCGGCGAGG-3′; *akirin1*, F: 5′-CCCTCCGACAAGTTGGCATA-3′, R: 5′-TAGCTTGTTGGCCTTGTCCC-3′; *rnf10*, F: 5′-GCTGGAGTATCTGTCTGCCT-3′, R: 5′-TCAGTGCAAATGGTCCCCTC-3′; *thrap5/med16*, F: 5′-CTGACCCGCATGATCCACAT-3′, R:5′-CTATTAGCCAGGTGGTCCGC-3′) and the Kapa SYBR Fast One-Step qRT-PCR Kit (Sigma–Aldrich, St. Louis, MO, U.S.A.) and the Rotor-Gene Real-Time PCR Detection System (Qiagen). A dissociation curve was run at the end of the reaction to ensure that only one amplicon was formed and that the amplicons denatured consistently in the same temperature range for every sample. The mRNA levels were normalized against human *β-actin* (F: 5′-CTCGCCTTTGCCGATCC-3′; R: 5′-CGCCCACATAGGAATCCTTC-3′) using the genNorm ΔΔ-Ct (ddCt) method as previously described [31]. Normalized *C*_t_ values were compared between test WT and *akirin2*-KO HL60 cells by Student’s *t* test with unequal variance (*P*<0.05; *n*=3 biological replicates).

### Proteomics data acquisition and analysis in human HL60 cells

Total proteins were extracted from WT and *akirin2* KO HL60 cells using the AllPrep DNA/RNA/Protein Mini Kit (Qiagen) and protein concentration was determined using the BCA Protein Assay with BSA (Sigma–Aldrich) as standard. Protein extracts (150 µg per sample, *n*=3 biological replicates) from each WT and KO cells were trypsin digested using the FASP Protein Digestion Kit (Expedeon Ltd., Abcam, Cambridge, U.K.) and sequencing grade trypsin (Promega, Madison, WI, U.S.A.) following manufacturer’s recommendations. The resulting peptides were finally desalted on to OMIX Pipette tips C18 (Agilent Technologies, Santa Clara, CA, U.S.A.), dried down and stored at −20 °C until mass spectrometry analysis. The desalted protein digests were resuspended in 2% acetonitrile, 5% acetic acid in water and analyzed by reverse-phase liquid chromatography mass spectrometry (RP-LC-MS/MS) using an ekspert™ nanoLC 415 system coupled online with a 6600 TripleTOF mass spectrometer (AB Sciex, Framingham, MA, U.S.A.) through information-cyclic data-independent acquisition (DIA) followed by sequential windowed data-independent acquisition of the total high-resolution mass spectra (SWATH)-mass spectrometry (MS). Three micrograms of each protein digest from the three biological replicates of WT and *akirin2* KO HL60 cells were used for the generation of the reference spectral ion library as part of the SWATH-MS analysis.

The peptides were concentrated in a 0.1 × 20 mm C18 RP precolumn (Thermo Fisher Scientific), and then separated using a 0.075 × 250 mm C18 RP column (New Objective, Woburn, MA, U.S.A.) operating at 0.3 µl/min. Peptides were eluted in a 120-min gradient from 5 to 30% solvent B in solvent A followed by 15-min gradient from 30 to 60% solvent B in solvent A (solvent A: 0.1% formic acid in water, solvent B: 0.1% formic acid in acetonitrile) and directly injected into the mass spectrometer for analysis. For IDA experiments, the mass spectrometer was set to scanning full spectra (350–1400 m/z) using 250 ms accumulation time per spectrum, followed by up to 50 MS/MS scans (100–1500 m/z). Candidate ions with a charge state between +2 and +5 counts per second above a minimum threshold of 100 were isolated for fragmentation. One MS/MS spectrum was collected for 100 ms, before adding those precursor ions to the exclusion list for 15 s (mass spectrometer operated by Analyst TF 1.6, AB Sciex). Dynamic background subtraction was turned off. MS/MS analyses were recorded in high-sensitivity mode with rolling collision energy on and a collision energy spread of 5. For SWATH quantitative analysis, six independent samples (three replicates from WT and *akirin2* KO HL60 cells, 5 µg each) were subjected to the cyclic DIA of mass spectra using the SWATH variable windows calculator (V 1.0, AB Sciex) and the SWATH acquisition method editor (AB Sciex), similar to established methods [32]. A set of 50 overlapping windows was constructed (containing 1 m/z for the window overlap), covering the precursor mass range of 400–1250 m/z. For these experiments, a 50-ms survey scan (350–1400 m/z) was acquired at the beginning of each cycle, and SWATH–MS/MS spectra were collected from 100–1500 m/z for 70 ms at high-sensitivity mode, resulting in a cycle time of 3.6 s. Collision energy for each window was determined according to the calculation for a charge +2 ion-centered upon the window with a collision energy spread of 15. To create a spectral library of all the detectable peptides in the samples, the IDA MS raw files were combined and subjected to database search in unison using ProteinPilot software v. 5.0.1 (AB Sciex) with the Paragon algorithm. Spectra identification was performed by searching against the Uniprot human proteome database (https://www.uniprot.org) (75074 entries in September 2020) with the following parameters: iodoacetamide cysteine alkylation, trypsin digestion, identification focus on biological modification and thorough ID as search effort. The detected protein threshold was set at 0.05. An independent false discovery rate (FDR) analysis, using the target–decoy approach provided by Protein Pilot (AB Sciex; https://sciex.com/products/software/proteinpilot-software), was used to assess the quality of identifications. Positive identifications were considered when identified proteins reached 1% of global FDR. For SWATH processing, up to ten peptides with seven transitions per protein were automatically selected by the SWATH Acquisition MicroApp 2.0 in the PeakView 2.2 software (AB Sciex; https://sciex.com/products/software/peakview-software) with the following parameters: 15 ppm ion library tolerance, 5 min XIC extraction window, 0.01 Da XIC width, and considering only peptides with at least 99% confidence and excluding those which were shared or contained modifications. However, to ensure reliable quantitation, only proteins that had three or more peptides available for quantitation were selected for XIC peak area extraction and exported for analysis in the MarkerView 1.3 software (AB Sciex; https://sciex.com/products/software/markerview-software). Global normalization was performed according to the Total Area Sums (TAS) of all detected proteins in the samples. The Student’s *t* test (*P*<0.05) was used to perform two-sample comparisons between the averaged TAS of all the transitions derived from each protein across the three replicate runs for each sample under comparison in order to identify proteins that were significantly differentially represented between WT and *akirin2* KO HL60 cells (Supplementary Data S3). GO annotations were obtained using Blast2GO software (http://www.blast2go.org; accessed on 20 September 2020). Heatmaps were prepared using the Heatmapper (http://www.heatmapper.ca/expression/) with average linkage and Euclidean distance measurement method. The raw proteomics data have been deposited at the ProteomeXchange Consortium (http://proteomecentral.proteomexchange.org) via the PRoteomics IDEntifications (PRIDE) partner repository with the dataset identifier PXD022073 and DOI: 10.6019/PXD022073.

### Network analysis of RNAseq transcriptomics data of human HL60 cells

A network is a set of nodes that are connected by edges. In the classic body of science devoted to food webs [33] or parasitic networks [34] nodes are the interacting organisms, and links between nodes represent the strength with which they interact. The direction and strength of the interaction has a weight such as the number of times a parasite has been found on a host [35]. The network construct proposed here is bipartite and metaphorical, meaning that a gene is the source node and the cell BP in which it is involved are the targets. Each gene was annotated with its role in a BP according to GO annotations. The network was built with a ‘source’ (the gene) and a ‘destination’ (the BPs in which each gene is involved). The link between source and destination has strength equivalent to the representation of the gene in WT and *akirin2* KO human HL60 cells. The degree of each node was then calculated according to either the representation of the gene or the sum of links reaching a BP (the destination). The network is directed and the edge linking both nodes has a weight, which is the representation of the gene. Since the expression profile of each gene changes in either WT or *akirin2* KO cells, it is therefore the indicator of its over- or under-representation. The weighted degree obtained from the sum of representation profiles of each link between genes and BPs is the fundamental brick of the network. All the calculations explained below were separately performed for the network of the sets of genes detected in either WT or *akirin2* KO cells. Centrality is a fundamental property of a network because it refers to nodes that connect other high score nodes. Therefore, genes with a high centrality can be regarded as pivotal parts of the network, while highly central processes can be considered as fundamental for the cell. We calculated the eigenvector centrality, a measure of the importance of a node in the ‘traffic’ between different nodes of a network. In our context, it is an indicator of the relative importance of a gene in WT versus *akirin2* KO cells. The rate of change of the eigenvector centrality between the networks built for both types of cells was calculated to capture the impact of the manipulation of the cells on the structure of the network. The focus was on the detection of significant changes in the centrality of each gene in wild versus knockout cells. Relationships between genes and BPs were built according to a graph representation and displayed using the Force Atlas 2 algorithm [36]. Clusters (i.e., modules of genes and BPs interacting more frequently among them than with the other members of the network) were obtained using the Lovaine algorithm [37]. All the work on the networks was done using the software Gephi 0.92 (www.gephi.org, accessed in September 2020) and disclosed in Supplementary Data S4.

### Protein–protein interaction network and functional enrichment analysis

The analysis was conducted with Akirin2 gene/protein targets using genes and proteins with the highest and lowest log2(*akirin2* KO/WT)-fold change after RNAseq and proteomics analyses with STRING (version 11.0; https://string-db.org/cgi/input?sessionId=bavpGR4XxxCp&input_page_show_search=off). STRING settings provided full network (the edges indicate both functional and physical protein associations) with the highest confidence (0.900). Based on available information, protein–protein interaction functional enrichment analysis was conducted for GO:BP, Kyoto Encyclopedia of Genes and Genomes (KEGG) pathways (https://www.genome.jp/kegg/), Reactome Pathways (RCTM; https://reactome.org) and local network cluster (STRING) Network Neighbor AL-2 tool.

### Recombinant Akirin2 proteins

Human recombinant Akirin2 (recAkirin2; Q53H80) produced in *Escherichia coli* (GenScript, Piscataway, NJ, U.S.A.) or in human kidney HEK293T cells (TP300881; OriGene, Rockville, MD, U.S.A.) was COOH-(Myc-(EQKLISEEDL)-DDK)-tagged and was used for the characterization of Akirin2–protein interactions. The expected molecular weight for Akirin2 is 22–25 kDa [10,38], but the recAkirin2-tagged molecular weight is approximately 30 and 60 kDa for the monomer and dimer, respectively [14].

### Western blot analysis of HL60 protein extracts

Total proteins were extracted from WT and *akirin2* KO HL60 cells using the AllPrep DNA/RNA/Protein Mini Kit (Qiagen) and protein concentration was determined using the BCA Protein Assay with BSA (Sigma–Aldrich) as standard. One microgram of recAkirin2-tagged protein produced in human cells was used as positive control. Positive control was run together with 100 µg of total proteins from WT and *akirin2* KO HL60 cells, separated by electrophoresis in a 12% sodium dodecyl sulfate (SDS)/polyacrylamide precast gel (ClearPage, Cole-Parmer, Vermon Hills, IL, U.S.A.) and transferred to a nitrocellulose blotting membrane (GE Healthcare Life Sciences, Pittsburgh, PA, U.S.A.). The membrane was blocked with 3% BSA in Tris-buffered saline (TBS; 150 mM NaCl, 50 mM Tris-HCl, pH 7.5) for 2 h at room temperature (RT) and washed three times with TBS-0.05% Tween 20. Akirin2-specific primary antibodies (ab130473, Abcam) were used for Akirin2 detection. Antibodies were diluted in TBS and incubated with membrane overnight at 4 °C. Goat anti-rabbit IgG (whole molecule) peroxidase antibodies (dilution 1:1000; Sigma–Aldrich) diluted in TBS with 3% BSA were used as secondary antibodies and incubated with the membrane for 2 h at RT. The membrane was finally washed six times with TBS-0.05% Tween 20, and immunoreactive proteins were visualized with chemiluminescence by incubating the membrane for 1 min with Pierce ECL Western blotting substrate (Thermo Fisher Scientific). For quantitation, gels were scanned with ImageJ (https://imagej.nih.gov/ij/index.html).

### Histone microarray analysis

EpiTriton Histone Peptide Arrays were used following manufacturer’s recommendations (Epicypher, Inc., Durham, NC, U.S.A.) with recombinant human Akirin2 produced in human cells and *Anaplasma phagocytophilum* heat shock protein 70 (HSP70) as negative control. The HSP70 was produced in *E. coli* using the Champion pET101 Directional TOPO expression kit (Invitrogen) as previously described [39]. Each protein was placed inside the histone array diluted in array buffer (phosphate-buffered saline, PBS, 5% BSA (w/v), 0.1% Tween-20) at 2 µM concentration. Proteins were incubated inside the array overnight at 4°C. Next day, arrays were washed with array buffer and incubated with protein-specific 1:500 dilution in array buffer of primary rabbit polyclonal antibodies for Akirin2 (ab130473, Abcam) and HSP70 [39] for 2.5 h at RT. Slides were washed in cold PBS and probed with a fluorescently labeled Alexa Fluor 635 goat anti-rabbit IgG secondary antibody (A31576; Invitrogen). Microarrays were scanned and analyzed using a Genepix Personal 4100A microarray scanner (Molecular Devices, LLC., San Jose, CA, U.S.A.) for measuring fluorescence in relative fluorescence units (RFUs) (Supplementary Data S6). Fluorescence was normalized against control empty spots (average of 87 technical replicates) and the values compared between Akirin2 and HSP70 by Student’s *t* test (*P*<0.05) and One-Way ANOVA (https://goodcalculators.com/one-way-anova-calculator/; *P*<0.05) tests (*n*=2 technical replicates; Supplementary Data S6).

### Corroboration of Akirin2–histone H3.1 peptide interaction by protein pull-down and Western blot analyses

Human recAkirin2 produced in *E. coli* and in human cells were used for interactions with synthetic peptide derived from human histone H3.1, H3 105–124 (amino acids 105–124, Ac-EDTNLCAIHAKRVTIMPKDI-Peg-K(Biot)-NH_2_), H3ac (amino acids 15–34 acetylated at Lys^18^, Lys^23^ and Lys^27^, Ac-APRK(Ac)^18^QLATK(Ac)^23^AARK(Ac)^27^SAPSTGG-Peg-Biot) and H3met (amino acids 1–20 dimethylated at Lys^14^, ARTKQTARKSTGGK(Me2)APRKQL-Peg-Biot) (Epicypher, Inc.). Dynabeads MyOne Streptavidin T1 (Invitrogen) were used following manufacturer’s instructions. Magnetic beads were incubated with the biotinylated H3.1 peptide for 1 h at RT with agitation. Then, supernatant was removed, and beads were blocked with 1 µg of biotin (Thermo Fisher Scientific, Waltham, MA, U.S.A.) for 30 min at RT with agitation. After incubation, supernatant was removed, and 1 µg protein was added and incubated for 2 h at RT with agitation. Beads were washed with PBS/0.1% BSA and protein–H3 complex were eluted using Laemmli sample buffer with β-mercaptoethanol. Samples were boiled for 5 min and the supernatants used for Western blot analysis. As negative control, the same procedure was performed using Dynabeads MyOne Streptavidin T1, but without incubation with histone H3.1. The recAkirin2 (1 µg) was used as positive control. Samples were separated by electrophoresis in a 12% SDS/polyacrylamide precast gel (ClearPage, Cole-Parmer, Vermon Hills, IL, U.S.A.) and transferred to a nitrocellulose blotting membrane (GE Healthcare Life Sciences). The membrane was blocked with 3% BSA in TBS (150 mM NaCl, 50 mM Tris/HCl, pH 7.5) for 2 h at RT and washed four times with TBS-0.05% Tween 20. Akirin2-specific primary antibodies (ab130473, Abcam) were used for Akirin2 detection. Antibodies were diluted in TBS and incubated with membrane overnight at 4 °C. Goat anti-rabbit IgG (whole molecule) peroxidase antibodies (dilution 1:1000; Sigma–Aldrich) diluted in TBS with 3% BSA were used as secondary antibodies and incubated with the membrane for 2 h at RT. The membrane was finally washed five times with TBS-0.05% Tween 20, and immunoreactive proteins were visualized with chemiluminescence by incubating the membrane for 2 min with Pierce ECL Western blotting substrate (Thermo Fisher Scientific) and with TMB Stabilized Substrate for Horseradish Peroxidase (Promega). For quantitation, Western blot immunoreactive proteins visualized with chemiluminescence were scanned with ImageJ (https://imagej.nih.gov/ij/index.html) to calculate normalized (maximum − minimum) intensity.

### Corroboration of Akirin2–histone H3 105–124 interaction by ITC

The interaction between histone H3 105–124 and recAkirin2 produced was studied by ITC in an Auto-iTC200 calorimeter (MicroCal, Malvern-Panalytical, Malvern, U.K.). Calorimetric experiments were performed by titrating H3.1 (200 μM) into Akirin2 or BSA (200 μM) in PBS at 25 °C. Each assay consisted of 19 × 2 µl injections, with a time-spacing of 150 s, a stirring speed of 750 rpm and a reference power of 10 μcal/s. From the thermogram (thermal power as a function of time), the binding isotherm (ligand-normalized heats as a function of the molar ratio) was obtained through integration of the individual heat effect associated with each injection. The affinity (equilibrium dissociation constant, *K*_d_ [µM]), enthalpy (ΔH [kcal/mol]) and stoichiometry (n) of binding were determined by nonlinear least-squares regression analysis employing a model that considered a single binding site in Origin 7.0 (OriginLab, Northampton, MA, U.S.A.).

### Characterization of Akirin2–IPs interactions in human HL60 cells

Some of the previously identified Akirin2 IPs [14] were selected for analysis of Akirin2–IP interactions in human HL60 cells. The HL60 cells were pelleted, washed with cold PBS and proteins were extracted by adding 1 ml of cold TBS supplemented with protease inhibitor cocktail (Complete Mini EDTA-free, Roche, Switzerland). Cells were disrupted passing through a 20 G needle and sonicated (3 min/cycle, 3 cycles, 25°C) until lysate was clear. Lysate was centrifuged at 12000×***g*** for 30 min at 4°C, after which the supernatant was collected, and protein concentration was determined using the BCA Protein Assay using BSA as the standard. The COOH-(Myc-(EQKLISEEDL)-DDK)-tagged recAkirin2 produced in human cells was used for interactions with the identified Akirin2–IPs, mediator of RNA polymerase II transcription subunit 16 (THRAP5; Q9Y2X0), phosphatidylinositol transfer protein-α isoform (PITPNA; Q00169), protein Wnt-2 (WNT2; P09544), and splicing factor 3a subunit 1 (SF3A1; Q15459). The U1 small nuclear ribonucleoprotein 70 kDa (SNRNP70; P08621) was used as protein negative control. Akirin2 was incubated with c-Myc magnetic beads (c-Myc-Tag IP/Co-IP kit, Thermo Fisher Scientific, Waltham, MA, U.S.A.) specific for the Akirin2 protein tag, for 30 min at RT with mixing following manufacturer’s recommendations. Then, supernatants were removed, and beads were incubated either with 200 or 400 µg of human HL60 cells protein lysate for 2 h at RT. A negative control was also performed incubating only Akirin2 with the c-Myc magnetic beads. After lysate incubation, beads were washed five times with TBS-Tween 20 and one more with ultrapure water. Immunoprecipitated complexes were eluted from the beads using 0.1 M Glycine pH 2 and 1 M Tris-HCl pH 8.2 was added until neutralization of pH. Supernatants derived from Akirin2-HL60 protein lysate and negative controls were used for a Dot Blot protein detection. Additionally, HL60 protein lysate itself and recAkirin2 were used as dots, together with PBS which was used as negative control. For the dot blot performance, samples were tested against the different Akirin2 IPs primary antibodies following the manufacturer’s recommendations and using the Bio-Dot Microfiltration Apparatus (Bio-Rad Laboratories, Inc., Hercules, CA, U.S.A.). First, dots were applied and filtrated by gravity through a nitrocellulose blotting membrane (GE Healthcare Life Sciences). Then, dots were blocked with 1% BSA in TBS and filtrated by gravity. After blocking step, two washes were performed using TBS-Tween 20 and filtrated though the membrane by vacuum application. Different IP-specific primary rabbit antibodies were used for PITPNA (rabbit ab96519, Abcam, 1:500), SF3A1 (rabbit ab69903, Abcam, 0.25 μg/ml), THRAP5 (rabbit ab130996, Abcam, 1:2000), SNRNP70 (rabbit VPA00459, Bio-Rad Laboratories, 1:1000) and WNT2 (rabbit ab27794, Abcam, 2 μg/ml). Antibodies were diluted in TBS and filtrated by gravity. Two washes were performed and filtrated by vacuum application. Goat anti-rabbit IgG (whole molecule) peroxidase antibody (Sigma–Aldrich) 1:1000 diluted in TBS with 1% BSA were used as secondary antibodies and filtrated through the membrane by gravity. Dots were finally washed two times with TBS-Tween 20, and immunoreactive proteins were visualized with chemiluminescence by incubating the membrane for 1 min with Pierce ECL Western blotting substrate (Thermo Fisher Scientific). Area and percent analysis of each chemiluminescence dot signal were obtained and analyzed using Fiji program (ImageJ 1.52n; https://downloads.tomsguide.com/ImageJ,0301-31184.html). Dot blot areas were compared with the dots of SNRNP70 negative control by Student’s *t* test with unequal variance (*P*<0.05; *n*=2 biological replicates).

### Akirin2 ‘distraction’ through histone H3.1 and H2A peptides transfection in human HL60 cells

Human HL60 cells (1 × 10^5^ cells/well) were placed in a 24-well plate. Cells were transfected with 1.5 μg per well of H3 105–124 peptide or the unrelated histone H2A (amino acids 1–17) peptide (Epicypher, Inc.) using the Pierce Protein Transfection Reagent Kit (Thermo Fisher Scientific). Peptides were diluted in HEPES (4-(2-hydroxyethyl)-1-piperazineethanesulfonic acid) buffer (10 mM HEPES, 150 mM NaCl, pH 7.0) and used as solvent in transfection reagent. Transfection reagent/peptide complexes were resuspended in serum-free medium and delivered to the cells. After 4 h of incubation at 37 °C, one volume of 20% serum-containing medium was added directly to the wells. As a transfection control, 1.5 μg per well of fluorescein isothiocyanate (FITC)-conjugated antibody (Thermo Scientific) was transfected following the same protocol. Negative control cells were transfected only with HEPES buffer. Cells were harvested after 72 h by slow centrifugation at 1200 rpm for 5 min. Cell pellets were washed with sterile PBS buffer and used for total RNA extraction using TriReagent (Sigma–Aldrich) following the manufacturer’s recommendations. Protein transfection was confirmed in positive control-transfected cells in comparison with untreated control cells by fluorescence microscopy. Cells were mounted in ProLong Antifade with 4′,6-diamidino-2-phenylindole (DAPI) reagent (Molecular Probes, Eugene, OR, U.S.A.) and examined using a Zeiss LSM 800 laser scanning confocal microscope (Carl Zeiss, Oberkochen, Germany) with a 63× oil immersion objective. Total RNA was used to characterize the mRNA levels of selected genes by qRT-PCR using gene-specific oligonucleotide forward (F) and reverse (R) primers (*ELANE*, F: 5′-CGTGGCGAATGTAAACGTCC-3′, R: 5′-CCCGTTGAGCTGGAGAATCA-3′; *LHX2*, F: 5′-GACCACTTCGGCATGAAGGA-3′, R: 5′-TGCCCACGCCATTGTAGTAG-3′) using the Luna Universal One-Step qRT-PCR Kit (New England Biolabs, Ipswich, MA, U.S.A.) and the CFX96 Touch RT-PCR Detection System (Bio-Rad, Hercules, CA, U.S.A.). A dissociation curve was run at the end of the reaction to ensure that only one amplicon was formed and that the amplicons denatured consistently at the same temperature range for every sample. The mRNA levels were normalized against human β-actin (F: 5′-CTCGCCTTTGCCGATCC-3′; R: 5′-CGCCCACATAGGAATCCTTC-3′) using the genNorm ΔΔ-Ct (ddCt) method. Normalized *C*_t_ values were compared between histone-transfected and control cells by unpaired *t* test (https://www.graphpad.com/quickcalcs/ttest1/?Format=C) (*P*<0.05; *n*=3 biological replicates).

## Results and discussion

### The Akirin2-related regulome reveals gene/protein targets and candidate functional complements

**Figure 2:**
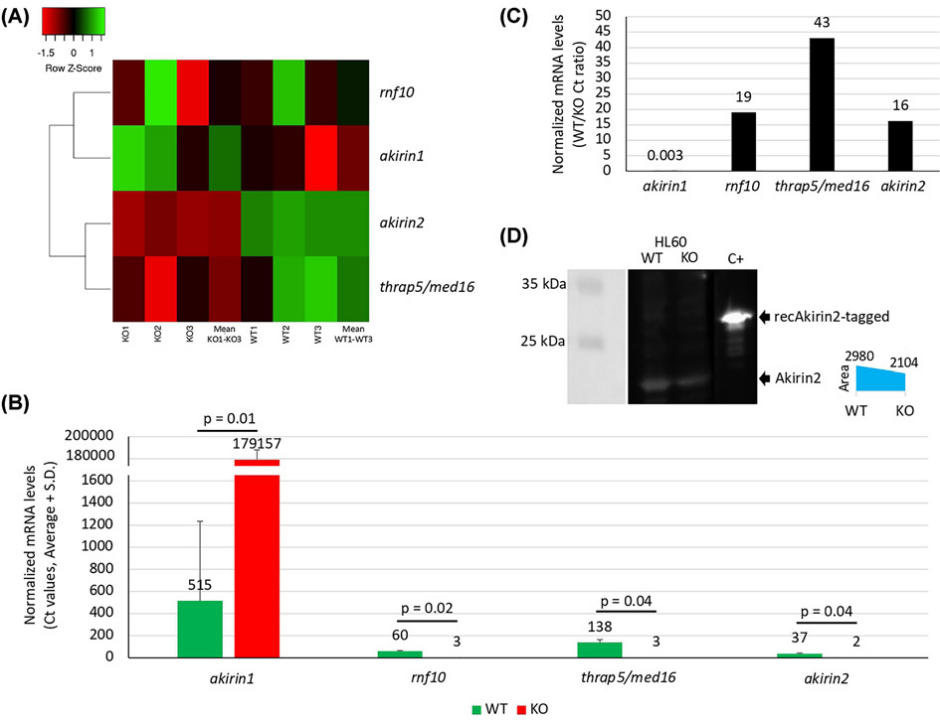
Analysis by qRT-PCR of selected genes in *akirin2* KO and WT HL60 cells Genes encoding for proteins identified as Akirin2 IPs and differentially regulated in response to *akirin2* KO were selected to validate transcriptomics data by qRT-PCR. (**A**) Heatmap of RNAseq results for selected genes. KO1-KO3 and WT1-WT3 mean values are shown. Heatmap was prepared with average linkage and Euclidean distance measurement method. (**B**) Normalized mRNA levels (average + S.D. *C*_t_ values) were compared between WT and KO cells by Student’s *t* test (*P*<0.05; *n*=3 biological replicates). (**C**) WT to KO ratio of normalized mRNA levels. (**D**) Western blot analysis of total protein extracts from WT and *akirin2* KO HL60 cells. One microgram of recAkirin2-tagged protein produced in human cells was used as positive control. Akirin2-specific primary antibodies were used for Akirin2 detection. For quantitation, gels were scanned with ImageJ (https://imagej.nih.gov/ij/index.html).

The RNAseq analysis of HL60 cells after *akirin2* KO in comparison with WT cells (*akirin2* KO/WT log2foldchange = −0.537; *P*=2.45E-21) identified a total of 17499 genes, of which 9863 (56%) were significantly regulated in response to *akirin2* KO (Supplementary Data S1). Of them, 4935 and 4928 were up- and down-regulated in response to *akirin2* KO, respectively. Then, 519 genes showing a KO/WT −2 < log2FoldChange > 2 were selected for further analysis (Supplementary Data S5). Genes encoding for proteins previously identified as Akirin2 IPs [14] and differentially regulated in response to *akirin2* KO were selected to validate transcriptomics data by qRT-PCR (Figure 2A–C). At the protein level, the proteomics analysis identified 2028 proteins of which 717 were differentially represented (227 over-represented and 490 under-represented proteins) in response to *akirin2* KO (Supplementary Data S3). Akirin2 protein levels were quantified by Western blot in *akirin2* KO and WT HL60 cells with a KO/WT log2foldchange = −0.514 (Figure 2D). Heatmaps of RNAseq and proteomics results evidenced clusters of up- and down-regulated genes in response to *akirin2* KO, which were not evident at the protein level (Supplementary Figure S1A–C).

Genes with the highest and lowest KO/WT log2foldchange were identified as those highly down-regulated and up-regulated by Akirin2, respectively (Table 1, Supplementary Figure S2A,B). Regulation of these genes may occur directly through Akirin2–IPs interactions or indirectly through genetic interactions or chromatin remodeling (Figure 1, Supplementary Data S1). Proteomics analysis revealed proteins with highest (over-represented in response to *akirin2* KO) or lowest (under-represented in response to *akirin2* KO) KO/WT log2foldchange different from those encoded by highly regulated genes, except for bone marrow proteoglycan (PRG2) that was up-regulated in response to *akirin2* KO at bot mRNA and protein levels (Table 1, Supplementary Figure S3A,B). Therefore, except for PRG2, and as shown before for the proteomics data (Supplementary Figure S1C), the levels of highly over- and under-represented proteins are possibly regulated at the post-transcriptional level by Akirin2 interactions with proteins such as actin-related protein 10 (ACTR10) and RING finger protein 10 (RNF10) [14].

**Table 1:** Akirin2 gene/protein targets

ID	Description [GO:BP classification] Protein–protein interaction functional enrichment (a) Network neighbor AL-2 (b) GO:BP (c) RCTM (d) KEGG	Log2-fold change (KO/WT)	*P*-value
Genes			
*IGLL1*	Immunoglobulin λ-like polypeptide 1 [Immune response] (a) Immunoglobulin complex, circulating, and Immunoglobulin V-type (b) Leukocyte migration (c) Cell surface interactions at the vascular wall (d) Not available	+6.4	0.0
*PRG2*	Bone marrow proteoglycan [Immune response] Not available	+5.8	0.0
*PRTN3*	Myeloblastin [Immune response] (a) Specific granule lumen, and azurophil granule lumen (b) Neutrophil degranulation (c) Neutrophil degranulation (d) Systemic lupus erythematosus	+4.1	0.0
*EPX*	Eosinophil peroxidase [Immune response] (a) Neutrophil-mediated killing of bacterium and Myeloperoxidase (b) Neutrophil degranulation (c) Neutrophil degranulation (d) Asthma	+4.0	0.0
*DUSP8*	Dual specificity protein phosphatase 8 [Dephosphorylation] (a) MAPK targets/nuclear events mediated by MAP kinases, and Mitogen-activated protein (MAP) kinase phosphatase (b) Stress-activated MAPK cascade (c) MAPK targets/nuclear events mediated by MAP kinases (d) MAPK signaling pathway	−8.1	8.2E-22
*CCL4*	C–C motif chemokine 4 [Immune response] (a) G α (i) signaling events (b) Chemokine-mediated signaling pathway (c) Interleukin-10 signaling (d) Cytokine–cytokine receptor interaction	−7.9	3.2E-241
*CD69*	Early activation antigen CD69 [Cellular response to drug] (a) TNFs bind their physiological receptors, and T-cell costimulation (b) Regulation of regulatory T-cell differentiation (c) RUNX1 and FOXP3 control the development of regulatory T lymphocytes (Tregs) (d) Autoimmune thyroid disease	−7.8	10E-14
*CCL4L2*	C–C motif chemokine 4-like [Immune and inflammatory response] (a) G α (i) signaling events (b) Chemokine-mediated signaling pathway (c) Chemokine receptors bind chemokines (d) Chemokine signaling pathway	−7.4	1.8E-169
*ULBP1*	UL16-binding protein 1 [Immune response] (a) Natural killer cell lectin-like receptor binding, and MHC class Ib receptor activity (b) Natural killer cell-mediated cytotoxicity (c) Immunoregulatory interactions between a Lymphoid and a non-Lymphoid cell (d) Natural killer cell-mediated cytotoxicity	−7.0	3.8E-8
Proteins			
ALB P02768	Albumin [Cell metabolism] (a) Regulation of Insulin-like Growth Factor (IGF) transport and uptake by IGF Binding Proteins (IGFBPs), and Formation of Fibrin Clot (Clotting Cascade) (b) Platelet degranulation (c) Post-translational protein phosphorylation (d) Fat digestion and absorption	+2.9	7.7E-5
PRG2 P13727	Bone marrow proteoglycan [Immune response] Not available	+2.7	0.001
LTF E7EQB2	Lactotransferrin [Immune response] (a) Specific granule lumen, and Vitamin B6 metabolism (b) Antimicrobial humoral response (c) Antimicrobial peptides (d) Not available	+2.6	6.4E-5
PDS5A Q29RF7	Sister chromatid cohesion protein PDS5 homolog A [Cell division] (a) Cohesin Loading on to Chromatin, and nuclear meiotic cohesin complex (b) Chromosome segregation (c) Establishment of Sister Chromatid Cohesion (d) Cell cycle	−3.5	0.007
PCK2 Q16822	Phosphoenolpyruvate carboxykinase [GTP], mitochondrial [Cell metabolism] (a) mRNA Splicing—Major Pathway (b) mRNA processing (c) mRNA Splicing—Major Pathway (d) Spliceosome	−2.8	3.8E-6
HTATSF1 O43719	HIV Tat-specific factor 1 [Transcription and virus replication] (a) mRNA Splicing—Major Pathway (b) mRNA splicing, via spliceosome (c) mRNA Splicing—Major Pathway (d) Spliceosome	−2.5	0.02

Genes were selected with −7.0 < log2(*akirin2* KO/WT)foldchange > 4.0. Proteins were selected with −2.0 < log2(*akirin2* KO/WT)foldchange > 2.0. Description and GO:BP classifications were obtained from UniProt (https://www.uniprot.org). The averaged reads derived from each gene/protein were compared between WT and *akirin2* KO HL60 cells by Student’s *t* test (*P*<0.05; *n*=3 biological replicates). Based on available information, protein–protein interaction functional enrichment analysis was conducted for GO:BP, KEGG pathways (https://www.genome.jp/kegg/), RCTM (https://reactome.org) and local network cluster (STRING) Network Neighbor AL-2 tool. Only entries with lowest FDR are shown. Full data are disclosed in Supplementary Data S1, 3 and 7.

The GO:BP classification and protein–protein interaction functional enrichment analysis at both mRNA and protein levels confirmed that Akirin2 is involved in the regulation of multiple BPs and identified regulation of cellular processes and immune response as a highly represented BPs in genes and proteins with positive and negative KO/WT log2FoldChange (Table 1, Supplementary Figures S2A,B, 3A and 4B, Supplementary Data S7).

The graph theory is used for the identification of networks of functionally interacting genes/proteins, which allow the characterization of cell transcriptome/proteome and regulome in response to stimuli such as pathogen infection [27,40]. Considering that Akirin2 is a transcription cofactor, in this study network analysis of transcriptome response to *akirin2* KO in human HL60 cells was used to identify genes and BPs with high centrality or relative importance in this process (Supplementary Figure S4). The network analysis of interacting genes and BPs identified immune effector process and anatomical structure formation involved in morphogenesis as the BPs with highest increase and decrease in relative importance after *akirin2* KO, respectively (Supplementary Data S4). Furthermore, while *akirin2* KO reduced to less than 1% the relative importance in the network of immune response and morphogenesis genes (*CLEC7A, RHOB, KLF2, CCL4*), the results identified immune response genes (*IGLL1, PRG2, PRTN3, EPX, CLC, CTSG*) as candidate Akirin2 functional complements based on their increase in relative importance in more than 1000% in response to *akirin2* KO (Supplementary Figure S4, Supplementary Data S4). Of them, *IGLL1, PRG2, PRTN3, EPX* were the most highly up-regulated genes in response to *akirin2* KO (Table 1). Furthermore, genes involved in other BPs in the regulation of cellular processes such as response to compounds and drugs, cell differentiation, apoptosis, RNA splicing, regulation of phosphatase activity and proteolysis (*AOC1, BEX1, SERPINB2, SNRPN, PPP1R27, PRSS57*) were also identified as candidate Akirin2 functional complements (Supplementary Data S4). *AZU1, PRG2* and *CTSG* were the genes with highest involvement in the control of multiple BPs and increased network relative importance in response to *akirin2* KO in human HL60 cells (Supplementary Figure S4, Supplementary Data S4). The *akirin1* mRNA levels increased by more than 300-fold in *akirin2* KO cells (Figure 2B,C) but was not identified as one of the most highly dysregulated genes/proteins nor with high centrality index in response to *akirin2* KO (Table 1, Supplementary Data S3 and S4). However, despite differences in the function between Akirin1 and Akirin 2 [1,41], we could not rule out the possibility that some of the changes in gene expression and protein representation after *akirin2* KO may be partly regulated by Akirin1.

These results suggested that humans as likely other metazoans with a conserved Akirin regulatory role evolved to guarantee the function of Akirin in multiple BPs with functional complements if required. These functional complements exercise their function in BPs in which Akirin2 is involved with high impact on the immune response, which plays a key role during evolution [7,42].

### Genes associated only with Akirin2 reveal BPs that may be regulated through unknown IPs, indirect (genetic) interactions or direct chromatin remodeling

**Figure 3:**
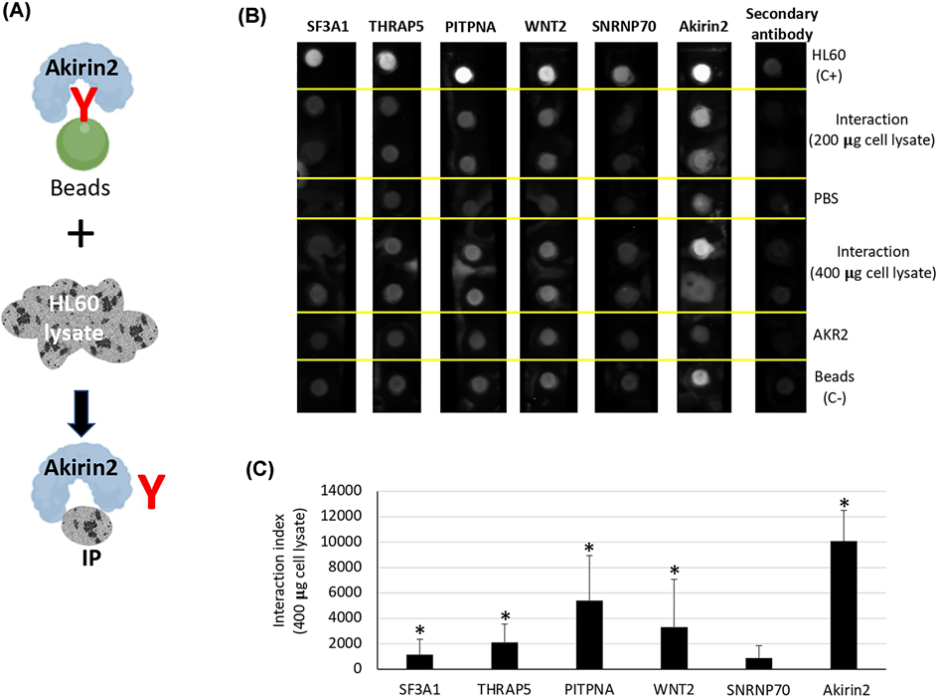
Corroboration of Akirin2–IP interactions in HL60 cells Some of the previously identified Akirin2 IPs [14] were selected for analysis of Akirin2–IP interactions in human HL60 cells. (**A**) Representation of the pull-down experiment using COOH-Myc-tagged human Akirin2 with c-Myc magnetic beads and HL60 cells lysate. (**B**) Akirin2 was incubated with HL60 lysate and immunoprecipitated with c-Myc magnetic beads specific for the Akirin2 protein Myc-tag. HL60 lysate and beads incubated only with HL60 lysate were used as positive (C+) and negative (C−) controls, respectively. PBS and recAkirin2 were also used as secondary controls. Akirin2-IPs were detected by mouse or rabbit antibodies specific for each protein and visualized by a dot blot analysis with anti-mouse or anti-rabbit secondary antibodies. (**C**) Dot blot areas of Akirin2–IPs dots were measured using ImageJ program and compared with the dots of SNRNP70, a protein used as negative control due to its low interaction with Akirin2, by Student’s *t* test with unequal variance (*P<0.05; *n*=2 biological replicates).

**Figure 4:**
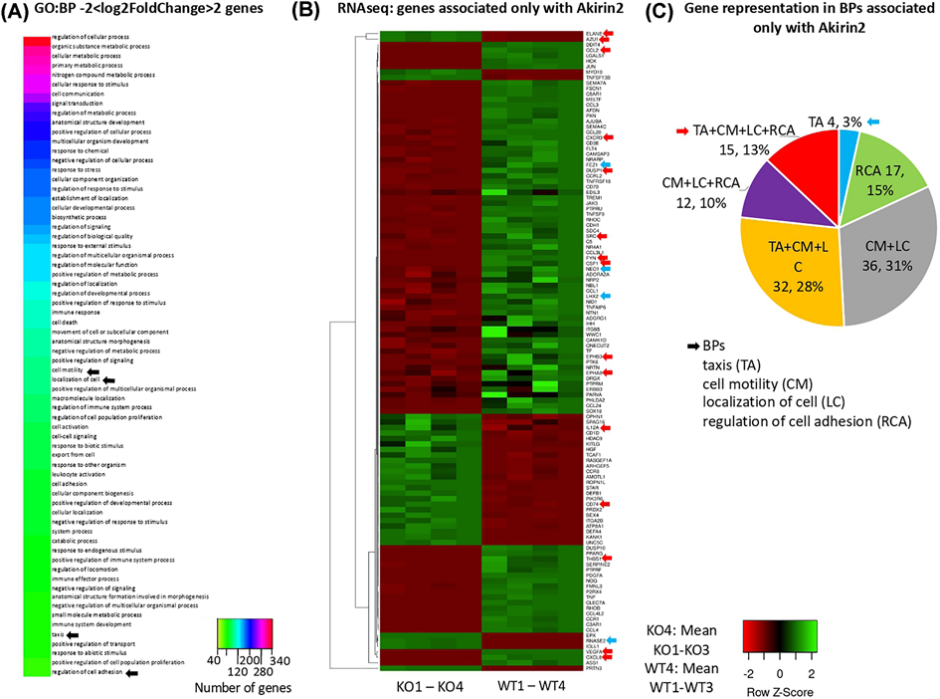
Differential expression and GO:BP of genes associated only with Akirin2 in response to *akirin2* KO in human HL60 cells (**A**) Heatmap of GO:BP annotations and number of genes with KO/WT −2 < log2foldchange > 2 on each BP. Black arrows indicate the BPs associated only with Akirin2. (**B**) Heatmap of RNAseq results for genes with KO/WT −2 < log2foldchange > 2 and associated only with Akirin2 (Supplementary Data S5). KO4 and WT4 correspond to KO1–KO3 and WT1–WT3 mean values, respectively. Red and blue arrows indicate genes associated with all four BPs or taxis alone, respectively. (**C**) Gene representation (*n*, %) in the four BPs, taxis (TA), cell motility (CM), localization of cell (LC) and regulation of cell adhesion (RCA) associated only with Akirin2. Heatmaps were prepared with average linkage and Euclidean distance measurement method.

To focus on BPs putatively regulated by Akirin2 alone, we first annotated the transcriptomics data for genes with KO/WT −2 < log2FoldChange > 2 in all the GO:BP classifications associated with known Akirin2 IPs, including data from BioGRID and Artigas-Jerónimo et al. [14] (Supplementary Data S5), of which some selected interactions were corroborated here in human HL60 cells (Figure 3A–C). Of the 68 annotated BPs, 64 BPs were associated with Akirin2 IPs and 4 BPs (taxis, cell motility, localization of cell, regulation of cell adhesion) were associated only with Akirin2 (Figures 1 and 4A,B and Supplementary Data S5). The most represented BPs at both gene (Figure 4A; Supplementary Data S5) and protein levels (Supplementary Data S3) were associated with regulation of cellular processes and immune response. The BPs associated only with Akirin2 correspond to processes regulating cell movement and attachment, immunity and chromatin remodeling (Figures 1 and 4A–C). A total of 96 genes were annotated in these BPs (Figure 4B), of which 4 and 17 were associated with taxis and regulation of cell adhesion unique BP categories, respectively while 15 genes were annotated in all 4 BPs (Figure 4C, Supplementary Data S5).

The Akirin/SUB have been implicated in host cell response to infection with the tick-borne pathogen, *Anaplasma phagocytophilum* [7]. Accordingly, of the 96 genes annotated in the four BPs associated only with Akirin2 (Figure 4B,C; Supplementary Data S5), three (P08246, neutrophil elastase, infected/control fold-change = 1.29; P09382, Galectin-1, infected/control fold-change = 2.42; P20160, Azurocidin, infected/control fold-change = 0.86) were previously reported to encode for proteins differentially represented in human HL60 cells in response to *A. phagocytophilum* infection [40].

Focusing on chromatin remodeling, six genes have been annotated in this GO:BP (GO:0006338) (Supplementary Data S5). Of them, *smarce1* (coding for SWI/SNF-related matrix-associated actin-dependent regulator of chromatin subfamily E member 1), *satb1* and *satb2* (coding for DNA-binding proteins SATB) were differentially regulated in response to *akirin2* KO (Supplementary Data S5). The gene coding for the actin-like protein 6 (ACL6A) that has been implicated in interactions with Akirin2 and chromatin remodeling [43] was identified as up-regulated in response to *akirin2* KO in HL60 cells (Supplementary Data S5). Additionally, a gene identified with 0.91% relative importance in the network analysis after *akirin2* KO, and thus likely regulated by Akirin2, was *KLF2* coding for Krueppel-like factor 2, which is involved in epigenetic regulation of gene expression [44,45] (Supplementary Data S4).

Taken together, these results identified BPs that may be regulated by Akirin2-mediated chromatin remodeling through interactions with known/unknown IPs, indirect (genetic) protein interactions for the activation of secondary factors and/or direct Akirin2–histone interactions (Figure 1).

### Akirin2 directly interacts with histone H3.1

Previous results support that Akirin2–IPs interactions regulate chromatin remodeling through ring finger protein 123 (RNF123) interaction with histone H3.1 [46] and ACL6A [43]. Akirin colocalizes with histone H3.1 and Brahma chromatin remodeling complex and was detected at an actively transcribed gene *locus* [17]. The results of our study showed that Akirin2 plays a role in the regulation of *smarce1, satb1* and *satb2*, which are involved in interactions with histone H3.1 and chromatin remodeling through uncharacterized IPs or indirect (genetic) interactions [47] (Supplementary Data S5).

**Figure 5:**
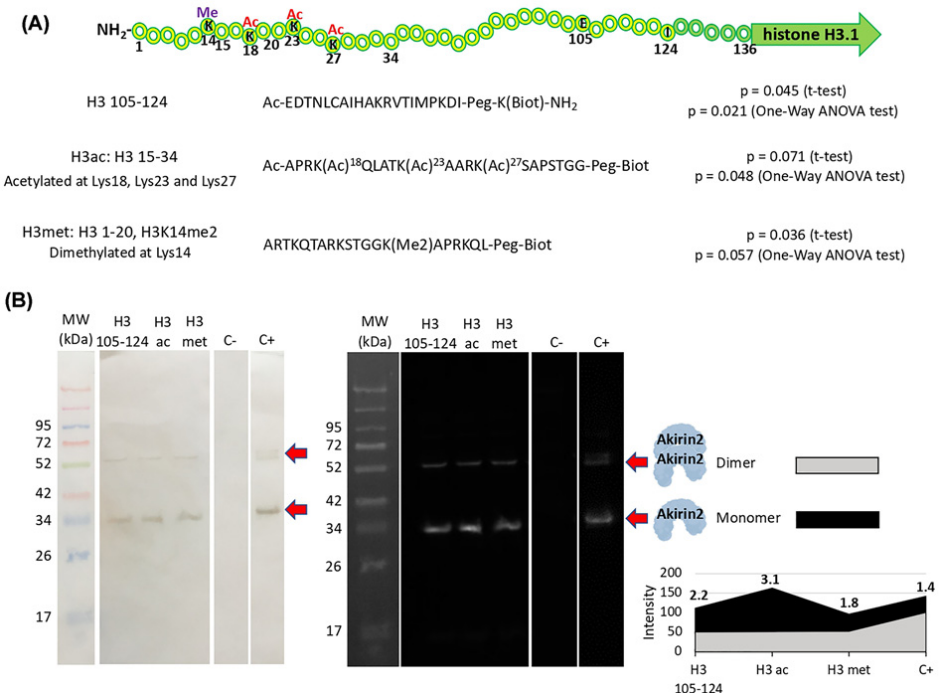
Identification of Akirin2–histone H3.1 physical interaction (**A**) Schematic representation of histone H3.1 protein used for the identification of Akirin2–histone interactions by recAkirin2 binding to a histone peptide array, which resulted in the identification of histone H3 105-124, H3ac and H3met as candidate interacting histone H3.1-derived peptides. Normalized fluorescence values were compared between Akirin2 and HSP70 by Student’s *t* test (*P*<0.05) and One-Way ANOVA (*P*<0.05) tests (*n*=2 technical replicates; Supplementary Data S6). Acetylated and methylated amino acids are shown. (**B**) Corroboration of the interactions between recAkirin2 (produced in insect cells) and histone H3 105–124, H3ac and H3met by *in vitro* protein pull-down and Western blot analysis with Akirin2-specific primary antibodies. Immunoreactive proteins were visualized with TMB (left panel) and chemiluminescence (right panel). Streptavidin beads incubated only with Akirin2 and recAkirin2 were included as negative (C−) and positive (C+) controls, respectively. For quantitation, Western blot immunoreactive proteins visualized with chemiluminescence were scanned with ImageJ (https://imagej.nih.gov/ij/index.html) to calculate normalized (maximum − minimum) intensity and Akirin2 monomer to dimer ratio (bold numbers).

However, is Akirin2 involved in direct interactions with histones? To address this question, a histone peptide array analysis was first used to identify potential Akirin2–histone interactions (Supplementary Data S6). The results identified three Akirin2–histone H3.1 interacting peptides, H3 105–124 (amino acids 105–124, Ac-EDTNLCAIHAKRVTIMPKDI-Peg-K(Biot)-NH_2_), H3ac (amino acids 15–34 acetylated at Lys^18^, Lys^23^ and Lys^27^, Ac-APRK(Ac)^18^QLATK(Ac)^23^AARK(Ac)^27^SAPSTGG-Peg-Biot) and H3met (amino acids 1–20 dimethylated at Lys^14^, ARTKQTARKSTGGK(Me2)APRKQL-Peg-Biot) (Figure 5A). These potential Akirin2–histone H3.1 interactions were then corroborated by *in vitro* protein pull-down and Western blot analysis with Akirin2-specific primary antibodies (Figure 5B). The quantitation of signal intensity of Akirin2–histone H3.1 interactions suggested differences in the role for Akirin2 monomer and dimer proteins (Figure 5B).

**Figure 6:**
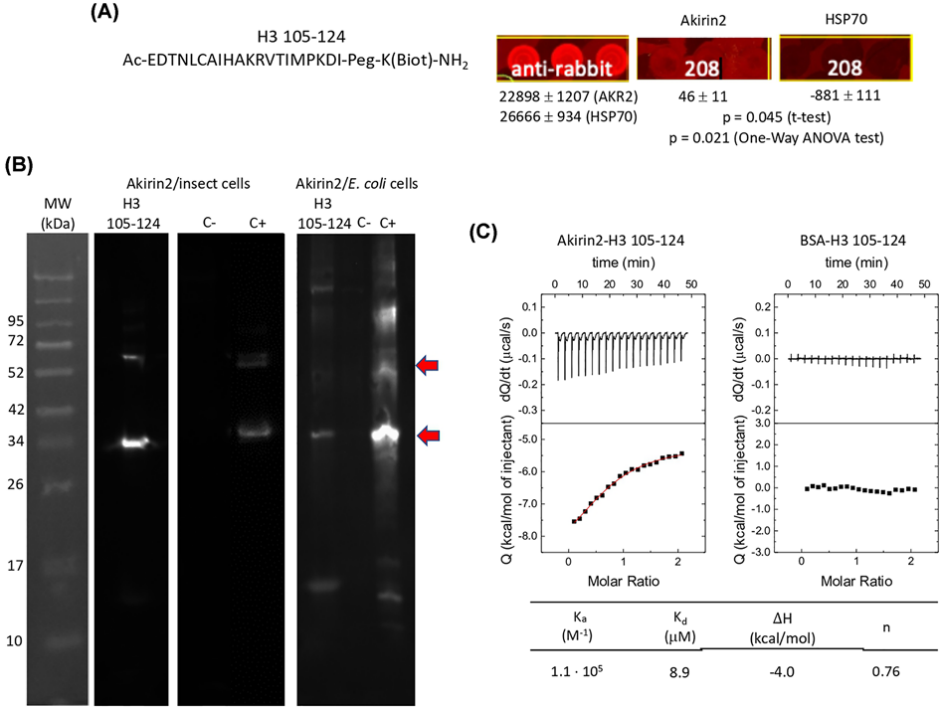
Characterization of Akirin2–histone H3 105–124 physical interactions (**A**) Akirin2–histone H3.1 interaction by recAkirin2 binding to a histone peptide array, which resulted in the identification of histone H3 105–124. Relative fluorescence is shown in RFU. (**B**) Corroboration of the interactions between recAkirin2 (produced in insect cells and *E. coli*) and histone H3 105–124 by *in vitro* protein pull-down and Western blot with Akirin2-specific primary antibodies. Immunoreactive proteins were visualized by chemiluminescence. Streptavidin beads incubated only with Akirin2 and recAkirin2 were included as negative (C–) and positive (C+) controls, respectively. (**C**) Corroboration of recAkirin2–histone H3 105–124 interactions by ITC. Calorimetric titrations of histone H3 105–124 into Akirin2 (left) and BSA (right). Upper plots show the thermogram and lower plots show the interaction isotherm. Non-linear regression data analysis allowed estimating the interaction parameters for Akirin2 (continuous red line): *K*_a_, association constant; *K*_d_, dissociation constant; ΔH, interaction enthalpy; n, fraction of active (binding-competent protein). No interaction was observed for BSA.

Epigenetic modifications of histone H3.1 at Lys amino acids has been involved in the regulation of transcription in different BPs and pathogen infection [48–51]. Nevertheless, we then focused on the most significant Akirin2−histone interaction with histone H3 105−124 as determined by the histone peptide array analysis (Figures 5A and 6A, Supplementary Data S6). The recAkirin2 (produced in insect cells and *E. coli*)-histone H3 105−124 interactions were then further corroborated by *in vitro* protein pull-down and Western blot analysis with Akirin2-specific primary antibodies (Figure 6B) and ITC (Figure 6C). Recently, the physical interaction between tick SUB and histone H4 but (amino acids 1–23 butyrylated at Lys 5, 8 and 12) was reported [52]. The results reported here demonstrated for the first time the physical Akirin2–histone H3.1 interaction, further supporting that Akirin2/SUB may be involved in chromatin remodeling for the regulation of gene expression.

### Towards functional implications of Akirin2–histone H3.1 interaction

To start addressing the functional implications of Akirin2-histone interactions, we focused on genes annotated in BPs associated only with Akirin2 and thus possibly regulated through Akirin2–H3.1 interaction. Two genes, *ELANE* (encoding neutrophil elastase, P08246, annotated in taxis, cell motility, localization of cell and regulation of cell adhesion BPs) and *LHX2* (encoding LIM/homeobox protein Lhx2, P50458, annotated in taxis BP) are up-regulated (*ELANE*, log2-fold change (KO/WT) = 2.3; *P*=0.0) or down-regulated (*LHX2*, Log2-fold change (KO/WT) = −5.0; *P*=6.4E-08) in human HL60 cells in response to *akirin2* KO, respectively (Supplementary Data S5). Of them, the transcriptional regulator *LHX2* has been previously implicated in chromatin binding (https://www.uniprot.org/uniprot/P50458).

**Figure 7:**
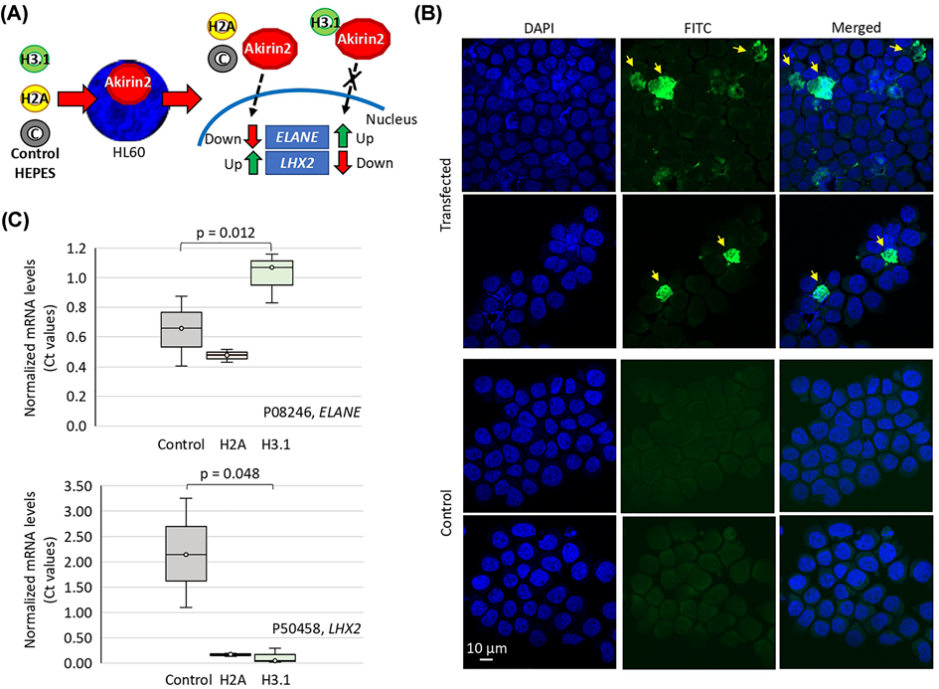
Functional characterization of Akirin2-histone H3.1 interaction (**A**) Akirin2 ‘distraction’ through histone H3.1 and H2A peptides transfection in human HL60 cells. Akirin2 interaction with H3.1 but not H2A or HEPES buffer control would interfere with Akirin2 regulation of *ELANE* and *LHX2* genes, which are up- and down-regulated, respectively, in response to *akirin2* KO in HL60 cells. (**B**) Transfection control of FITC-conjugated antibody. Protein transfection was confirmed (yellow arrows) in positive control-transfected cells in comparison with untreated control cells by fluorescence microscopy. (**C**) Total RNA was used to characterize the mRNA levels of *ELANE* and *LHX2* selected genes by qRT-PCR. The mRNA levels were normalized against human β-actin and normalized *C*_t_ values were compared between histone-transfected and control cells by unpaired *t* test (*P*<0.05; *n*=3 biological replicates).

The experimental approach was designed to ‘distract’ Akirin2 through interactions with transfected histones preventing its translocation to the nucleus and chromatin remodeling to exert gene regulation (Figure 7A,B). The results showed that transfection of H3.1 but not the unrelated H2A or HEPES buffer affected gene expression (Figure 7C). As expected for a possible role of Akirin2−H3.1 interaction in the regulation of these genes, the results in H3.1-transfected HL60 cells reproduced those obtained after *akirin2* KO.

These results provided evidence for the possible role of Akirin2-H3.1 interaction in the regulation of gene expression in human HL60 cells. Based on these results and previous findings on Akirin2 regulome and interactome [7,14,22], our hypothesis is that Akirin2 interacts with H3.1 without stimuli and selectively regulates gene expression through modulation of Akirin2 interactome to regulate various BPs in a biological context, cell type and stimulus-dependent manner.

## Conclusions

**Figure 8:**
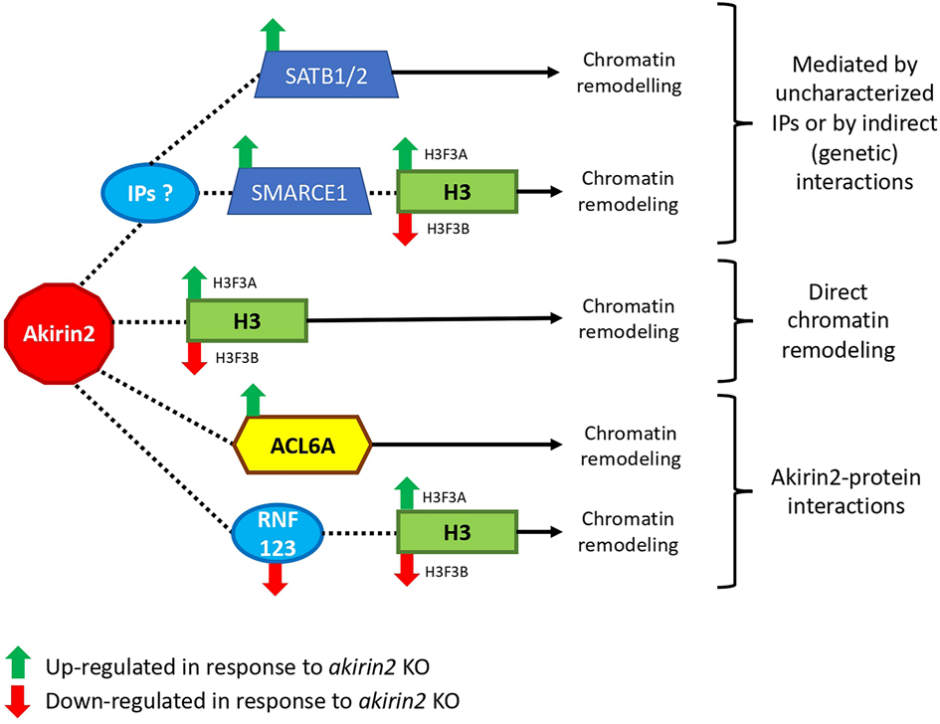
Role of Akirin2 in epigenetic regulation through chromatin remodeling Identified differentially regulated genes in response to *akirin2* KO (green arrow, up-regulated; red arrow, down-regulated) and implicated in interactions with Akirin2 and chromatin remodeling. We propose that chromatin remodeling is regulated by Akirin2 through interactions with known/uncharacterized IPs, indirect (genetic) protein interactions for the activation of secondary factors and direct interactions with histone H3.1.

In conclusion, the results of the present study together with previous reports support that Akirin2 plays a key role in host–pathogen interactions by regulating gene expression through chromatin remodeling by direct/indirect interactions with other regulatory factors and direct physical interactions with histone H3.1 (Figure 8). The identification of Akirin2 functional complements supports the key role of this regulatory protein throughout the metazoan [1,2,7], which resulted in evolutionary adaptations to complement its function if required. The results showed that Akirin2 do not only act as a link between a variety of transcription factors and major remodelers, but also physically interacts with histone H3.1 with possible functional implications in the regulation of gene expression. These results were obtained in the HL60 cells used as a model of neutrophils due to its role in innate immunity, but Akirin2–histone interactions should be explored in other cells lines and primary cells. Future experiments should be directed to functionally characterize how Akirin2–histone H3.1 interaction regulates transcription and identified BPs at the transcriptome/regulome level and in response to stimuli such as pathogen infection. Questions regarding functional differences in the role of Akirin2 monomer and dimer proteins and the possibility to reverse the Akirin2 phenotype by knocking down H3.1 should be addressed.

Based on results with tick SUB–histone interactions [52], these results provided additional support for the use of Akirin family proteins as protective vaccine antigens for the control of ectoparasite infestations and pathogen infection/transmission [7]. Targeting key regulatory proteins translates into reduced insect/tick fitness and reproduction, which will ultimately result in reduction in ectoparasite populations and transmitted diseases [7,14].

## Supplementary Material

Supplementary Figures S1-S4

Supplementary Data S1-S7

## Data Availability

RNAseq transcriptomics data in human HL60 cells can be found at NCBI under study name, ‘Effect of Akirin2 knockdown on the mRNA profile of human HL60 cells’ and GEO accession number: GSE158885 (https://www.ncbi.nlm.nih.gov/geo/query/acc.cgi?acc=GSE158885). The raw proteomics data in human HL60 cells can be found at the ProteomeXchange Consortium (http://proteomecentral.proteomexchange.org) via the PRoteomics IDEntifications (PRIDE) partner repository with the dataset identifier PXD022073 and DOI: 10.6019/PXD022073.

## Open Access

Open access for this article was enabled by the participation of CSIC Instituto de Investigacion en Recursos Cinegéticos in an all-inclusive *Read & Publish* pilot with Portland Press and the Biochemical Society under a transformative agreement with CSIC.

## Abbreviations

BP: biological process
DIA: data-independent acquisition
FDR: false discovery rate
GO: gene ontology
H: histone
H3: Histone H3
HSP70: heat shock protein 70
IP: Akirin-interacting protein
ITC: isothermal titration calorimetry
KO: knockdown
NF-κB: nuclear factor κ-light-chain-enhancer of activated B cells
PBS: phosphate-buffered saline
rec: recombinant
RNAseq: RNA sequencing
RT: room temperature
SDS: sodium dodecyl sulfate
SUB: subolesin
TAS: Total Area Sums
TLR: Toll-like receptor
TNF: tumor necrosis factor
WT: wildtype
