# Supplementary material for: Function of cofactor Akirin2 in the regulation of gene expression in model human Caucasian neutrophil-like HL60 cells
Source: Biosci Rep. 2021 Jul 22;41(7):BSR20211120. doi: 10.1042/BSR20211120 (PMC8298264; doi:10.1042/BSR20211120)
Supplement: Supplementary Figures S1-S4 [file BSR-2021-1120_supp.pdf]

Function of cofactor Akirin2 in the regulation of gene expression in model human Caucasian neutrophil-like HL60 cells

Sara Artigas-Jerónimo, Margarita Villar, Agustín Estrada-Peña, Adrián Velázquez-Campoy, Pilar Alberdi, José de la Fuente

Supplementary information

Supplementary Fig. S1. Differential gene expression and protein representation in response to *akirin2* KO in HL60 cells.
Supplementary Fig. S2. Protein-protein interactions network for RNAseq data.
Supplementary Fig. S3. Protein-protein interactions network for proteomics data.
Supplementary Fig. S4. Network analysis of genes and BPs in response to *akirin2* KO in HL60 cells.
Supplementary Data S2. Quality control assessment of RNAseq. Results for *akirin2* ENSG00000135334 and *ACTB* ENSG00000075624 were included as examples.

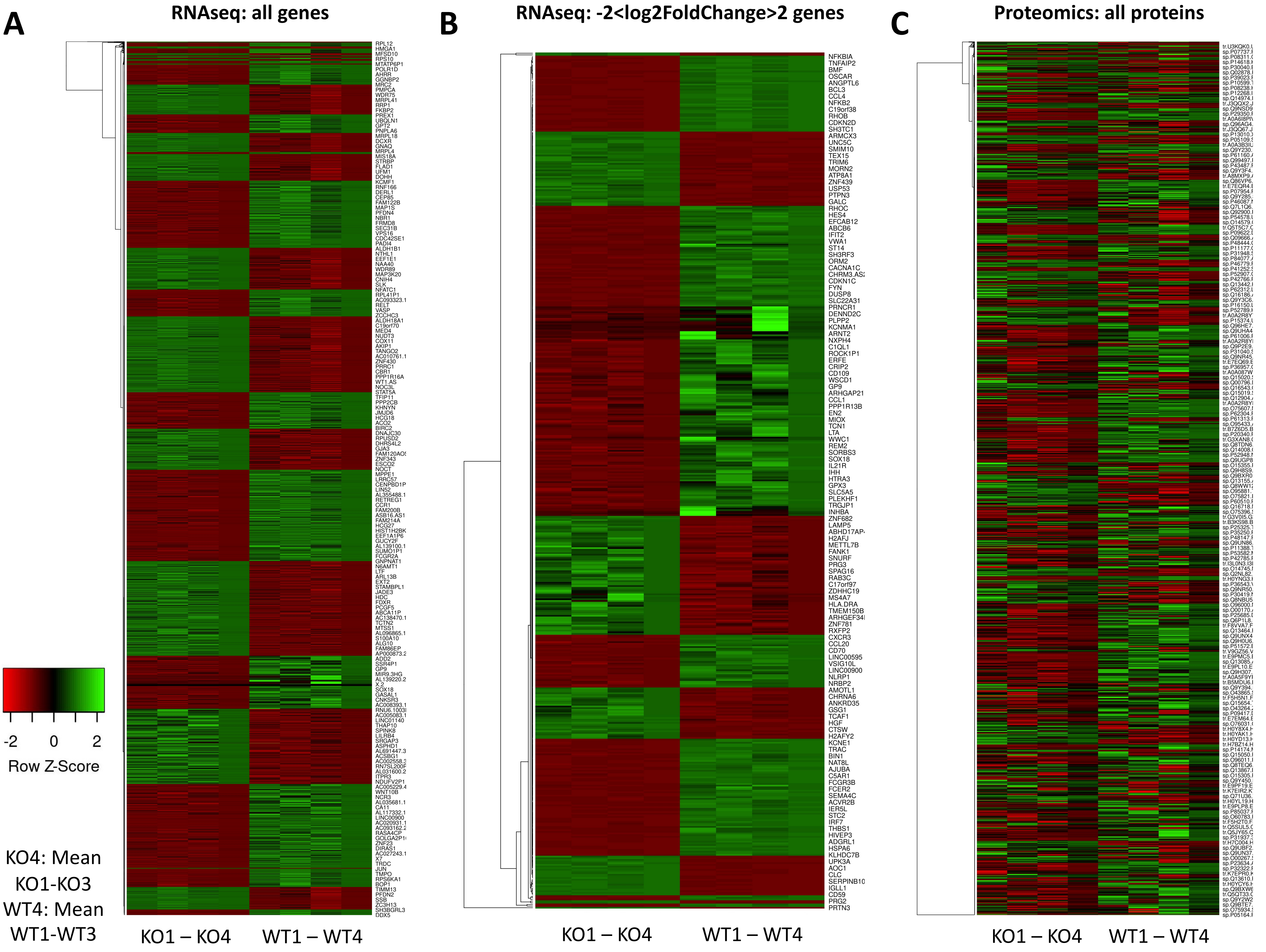
A
RNAseq: all genes
B
RNAseq: -2<log2FoldChange>2 genes
C
Proteomics: all proteins
-2
0
2
Row Z-Score
KO4: Mean
KO1-KO3
WT4: Mean
WT1-WT3
KO1 – KO4
WT1 – WT4
KO1 – KO4
WT1 – WT4
KO1 – KO4
WT1 – WT4

Supplementary Fig. S1. Differential gene expression and protein representation in response to *akirin2* KO in HL60 cells. (A) Heatmap of RNAseq results for all identified genes (Supplementary Data 1). (B) Heatmap of RNAseq results for genes with KO/WT -2<log2FoldChange>2 (Supplementary Data 5). (C) Heatmap of proteomics results for all identified proteins (Supplementary Data 3). KO4 and WT4 correspond to KO1-KO3 and WT1-WT3 mean values, respectively. Heatmaps were prepared with average linkage and Euclidean distance measurement method.

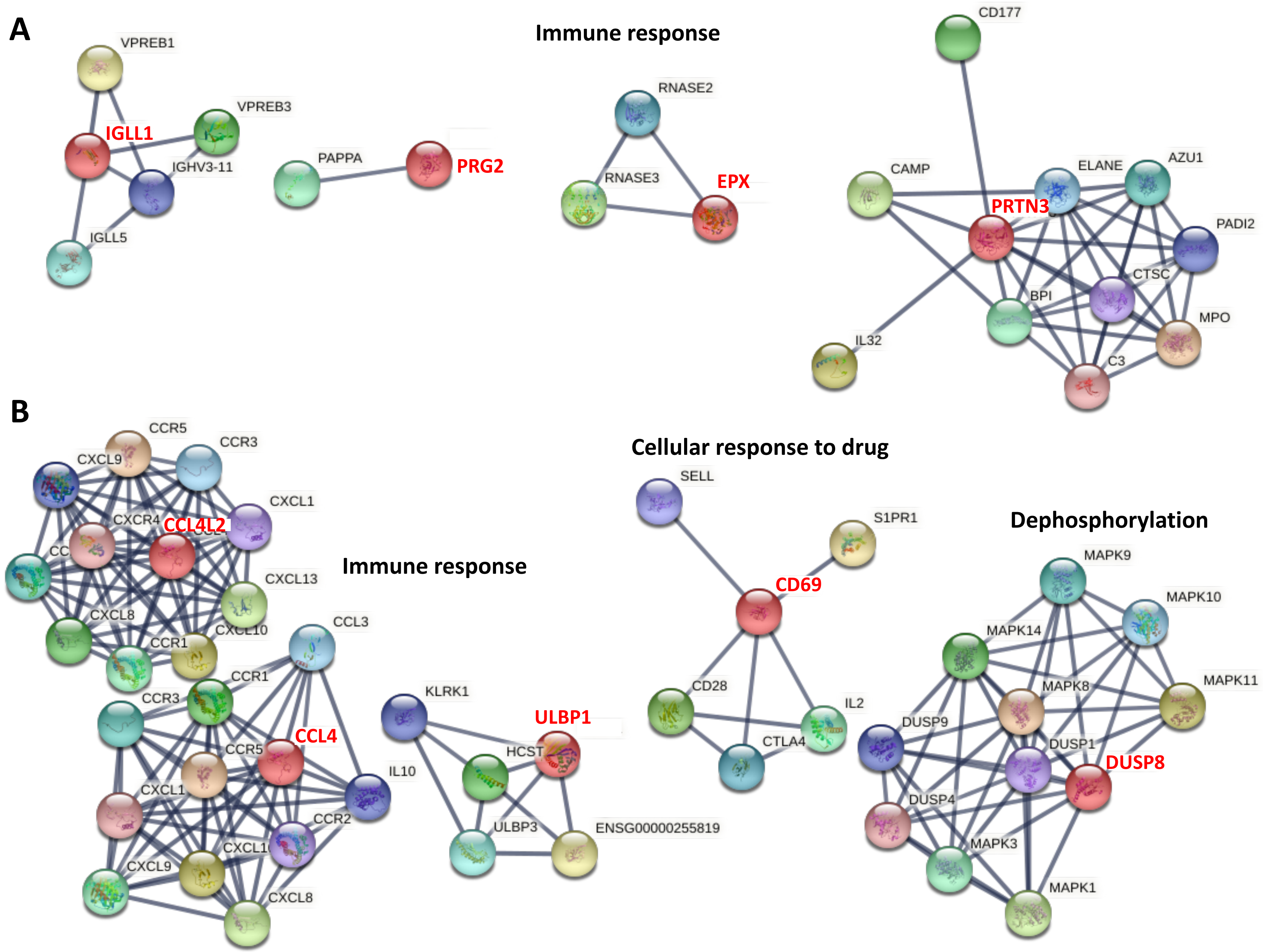
A
Immune response
VPREB1
IGLL1
VPREB3
IGHV3-11
IGLL5
PAPPA
PRG2
RNASE2
RNASE3
EPX
CD177
CAMP
ELANE
AZU1
PRTN3
PADI2
CTSC
BPI
MPO
IL32
C3
B
Cellular response to drug
Immune response
Dephosphorylation
CCR5
CCR3
CXCL9
CXCL1
CXCR4
CCL4L2
CXCL13
CXCL8
CXCL10
CCR1
CCL3
KLRK1
ULBP1
CCL4
HCST
IL10
CCR2
ULBP3
ENSG00000255819
SELL
S1PR1
CD69
CD28
IL2
CTLA4
MAPK9
MAPK10
MAPK14
MAPK11
MAPK8
DUSP9
DUSP1
DUSP8
DUSP4
MAPK3
MAPK1

Supplementary Fig. S2. Protein-protein interactions network for RNAseq data. The analysis was conducted with Akirin2 gene targets using genes with the (A) highest and (B) lowest log2(*akirin2* KO/WT)-fold change after RNAseq analysis with STRING. STRING settings provided full network (the edges indicate both functional and physical protein associations) with the highest confidence (0.900). Input genes (Table 1) are highlighted in red. The GO:BP classifications for different interactions are shown.

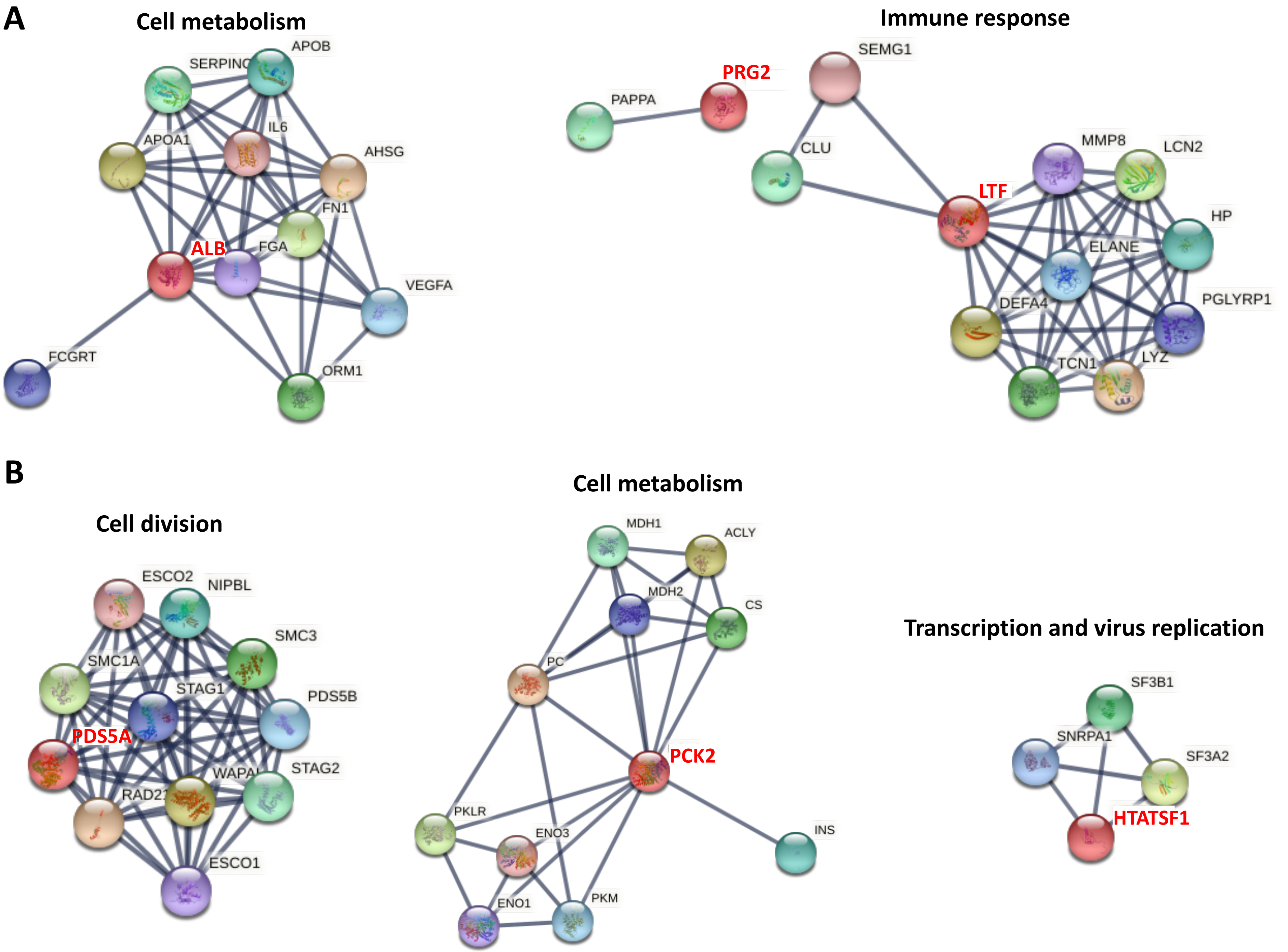

A
Cell metabolism
SERPINC
APOB
IL6
APOA1
AHSG
FN1
ALB
FGA
VEGFA
FCGRT
ORM1
Immune response
SEMG1
PRG2
PAPPA
CLU
MMP8
LCN2
LTF
HP
ELANE
DEFA4
PGLYRP1
TCN1
LYZ
B
Cell metabolism
Cell division
MDH1
ACLY
MDH2
CS
ESCO2
NIPBL
SMC3
SMC1A
STAG1
PDS5B
PDS5A
WAPAL
STAG2
RAD21
ESCO1
PC
PCK2
PKLR
ENO3
ENO1
PKM
INS
Transcription and virus replication
SF3B1
SNRPA1
SF3A2
HTATSF1

Supplementary Fig. S3. Protein-protein interactions network for proteomics data. The analysis was conducted with Akirin2 protein targets using proteins with the (A) highest and (B) lowest log2(*akirin2* KO/WT)-fold change after proteomics analysis with STRING. STRING settings provided full network (the edges indicate both functional and physical protein associations) with the highest confidence (0.900). Input proteins (Table 1) are highlighted in red. The GO:BP classifications for different interactions are shown.

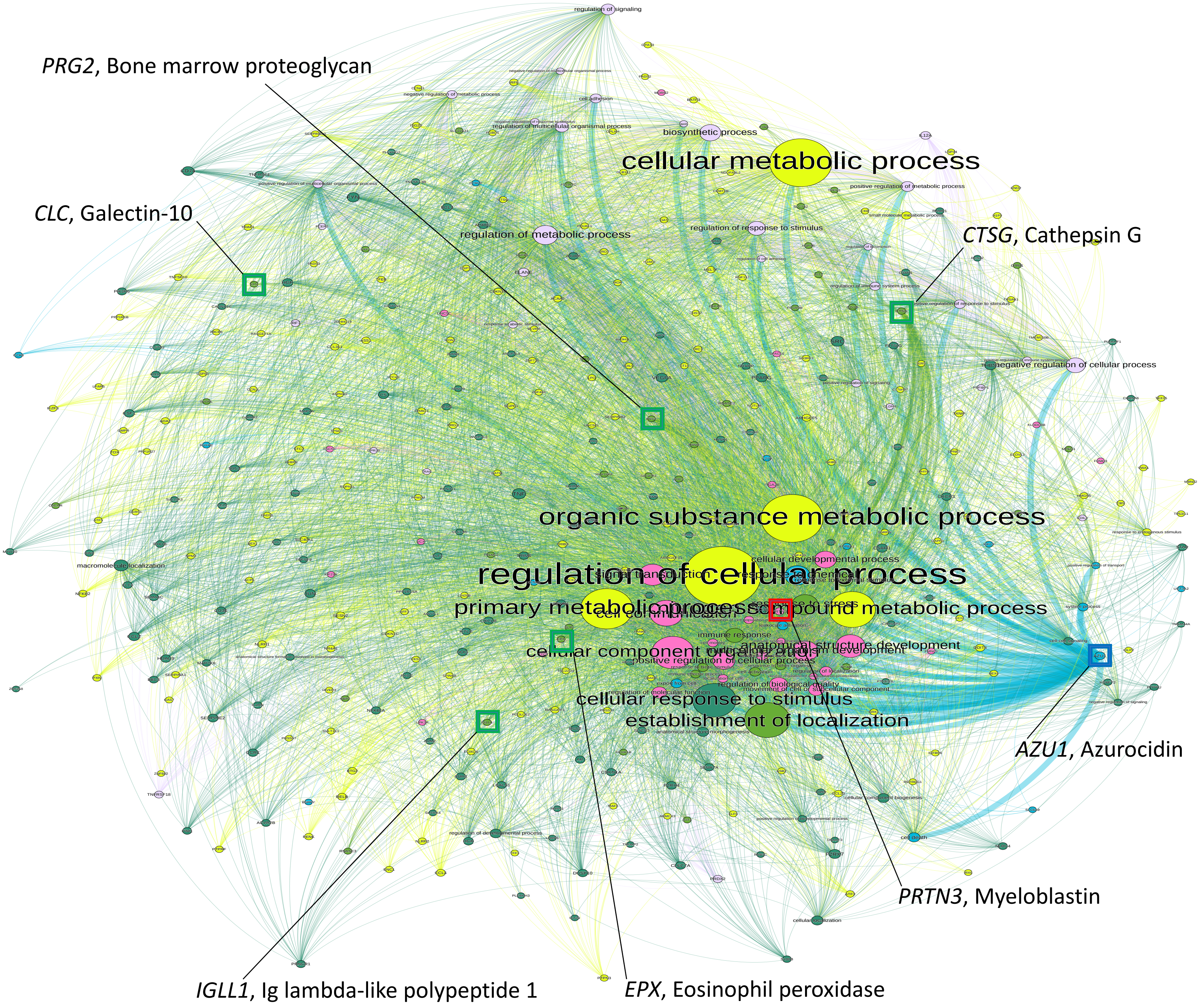

PRG2, Bone marrow proteoglycan
CLC, Galectin-10
CTSG, Cathepsin G
AZU1, Azurocidin
PRTN3, Myeloblastin
IGLL1, Ig lambda-like polypeptide 1
EPX, Eosinophil peroxidase
regulation of signaling
cell adhesion
biosynthetic process
regulation of multicellular organismal process
cellular metabolic process
positive regulation of metabolic process
regulation of metabolic process
regulation of response to stimulus
negative regulation of cellular process
organic substance metabolic process
cellular developmental process
regulation of cellular process
primary metabolic process
compound metabolic process
cellular component organization
anatomical structure development
positive regulation of cellular process
cellular response to stimulus
establishment of localization
macromolecule localization
immune response
regulation of biological quality
cell death
cellular component biogenesis
regulation of developmental process
cellular localization

Supplementary Fig. S4. Network analysis of genes and BPs in response to *akirin2* KO in HL60 cells. Relationships between genes and BPs built according to a graph representation and displayed using the Force Atlas 2 algorithm. Clusters (i.e., modules of genes and BPs interacting more frequently among them than with the other members of the network) are presented by colors and obtained by the Louvaine algorithm. The network is not nested because most of the genes are interacting simultaneously with different BPs. The size of each node is proportional to its importance in the network (i.e., "Regulation of cellular processes" is a very prominent feature of the network). Links among the circles are the interacting genes and BPs, and its width is proportional to the relative force of the interaction. The results highlight immune response genes (*IGLL1, PRG2, PRTN3, EPX, CLC, CTSG*) as candidate Akirin2 functional complements, and genes *AZU1*, *PRG2* and *CTSG* that showed highest involvement in the control of multiple BPs and increased network relative importance in response to *akirin2* KO in human HL60 cells.

Supplementary Data 2. Quality control assessment of RNAseq

file:///Users/josedelafuente1/Documents/Projects/Spain/PLAN%20NACIONAL/2016/Synthego/Report/DE-Report/Control-vs-Treatment_report.html

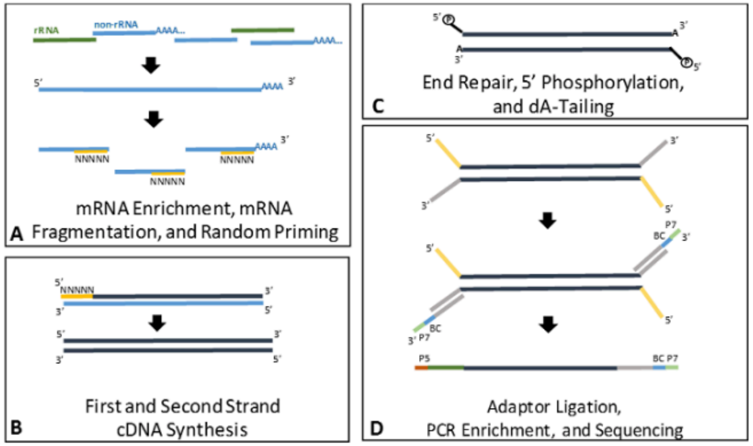

mRNA sequencing via polyA selection

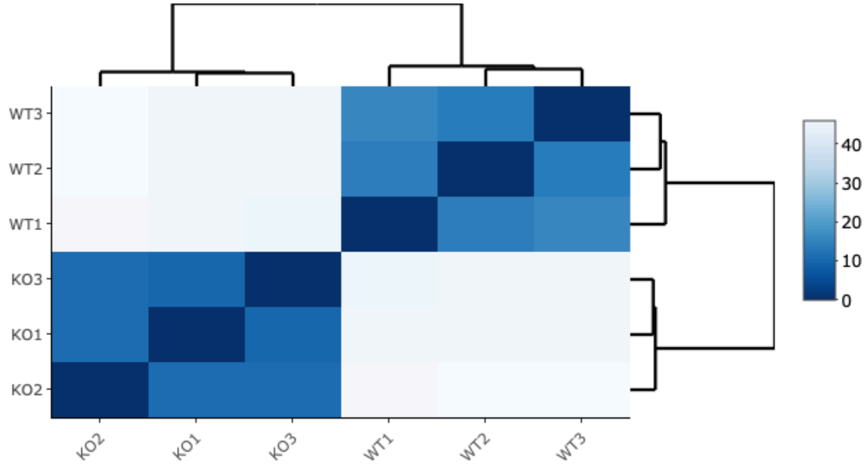

Sample similarity assessment. Heatmap of sample-to-sample distance

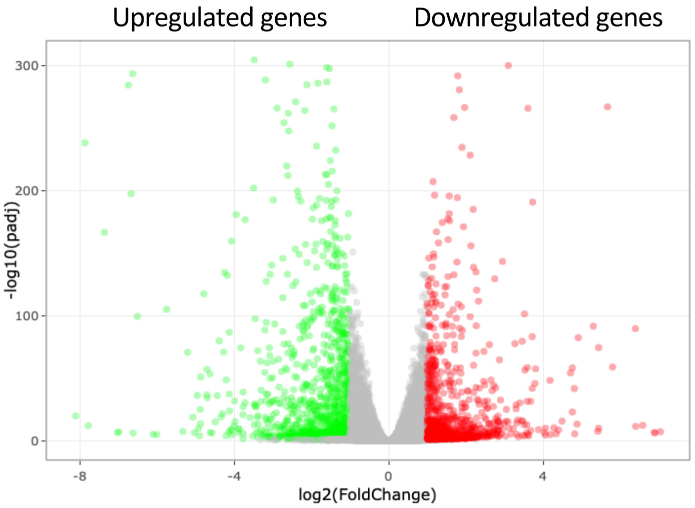

Global transcriptional change. Volcano plot

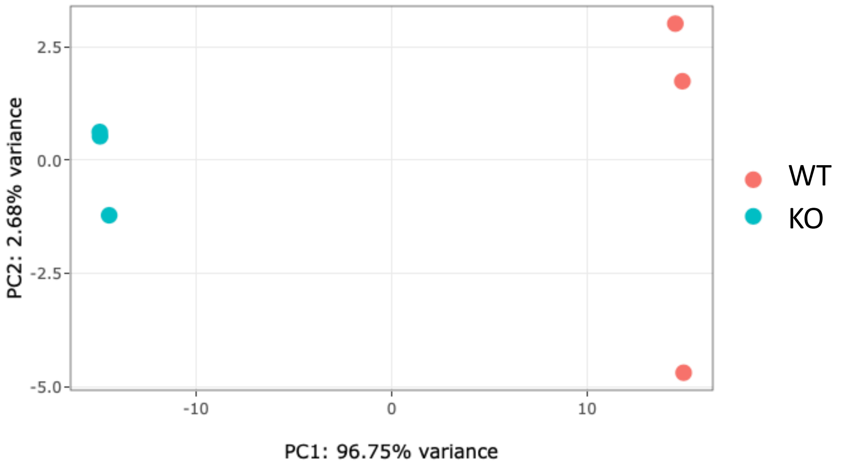

Sample similarity assessment. Principal component analysis

Gene: AKIRIN2 ENSG00000135334 http://www.ensembl.org/Homo_sapiens/Gene/Summary?g=ENSG00000135334;r=6:87674860-87702233

geneID	chromosome	start	end	total_exons	exon_changes
ENSG00000135334	6	87675072	87702209	7	4

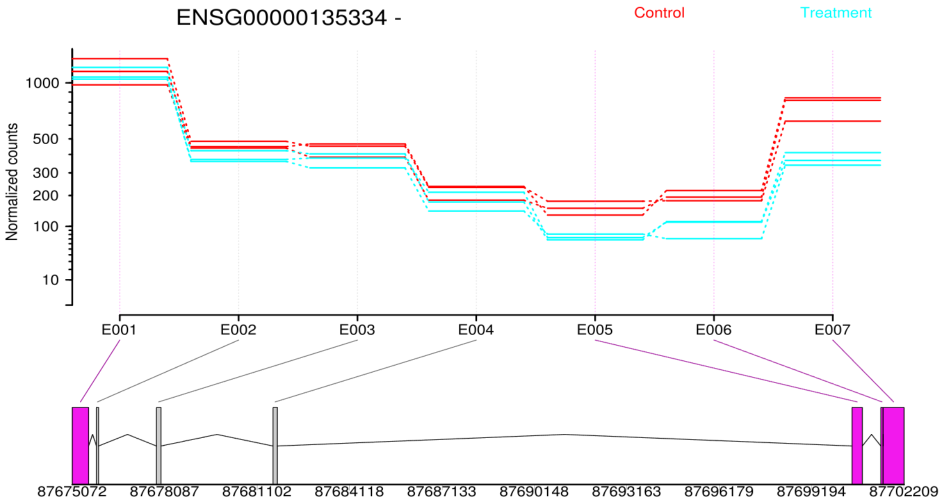

groupID	featureID	exonBaseMean	dispersion	pvalue	padj	seqnames	start	end	width	strand	Control	Treatment	log2fold_Treatment_Control
ENSG00000135334	E001	1118.9833	0.006	0.000	0.001	6	87675072	87675607	536	-	47.279	51.513	0.124
ENSG00000135334	E002	420.7398	0.001	0.032	0.142	6	87675860	87675931	72	-	32.090	33.271	0.052
ENSG00000135334	E003	401.2903	0.001	0.020	0.103	6	87677818	87677967	150	-	31.232	32.601	0.062
ENSG00000135334	E004	197.6717	0.007	0.551	0.788	6	87681620	87681763	144	-	22.765	23.303	0.034
ENSG00000135334	E005	114.4487	0.008	0.000	0.004	6	87700510	87700846	337	-	19.468	15.398	-0.338
ENSG00000135334	E006	148.1573	0.007	0.000	0.002	6	87701450	87701525	76	-	21.809	17.540	-0.314
ENSG00000135334	E007	567.7599	0.011	0.000	0.000	6	87701526	87702209	684	-	40.147	32.785	-0.292

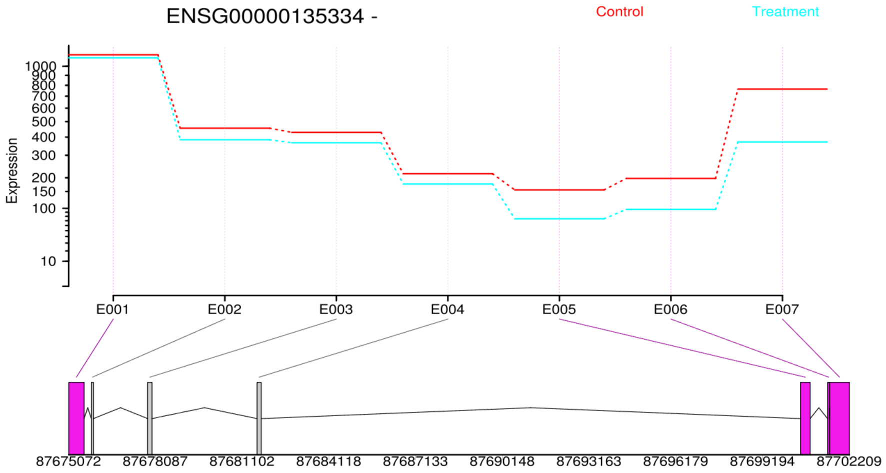

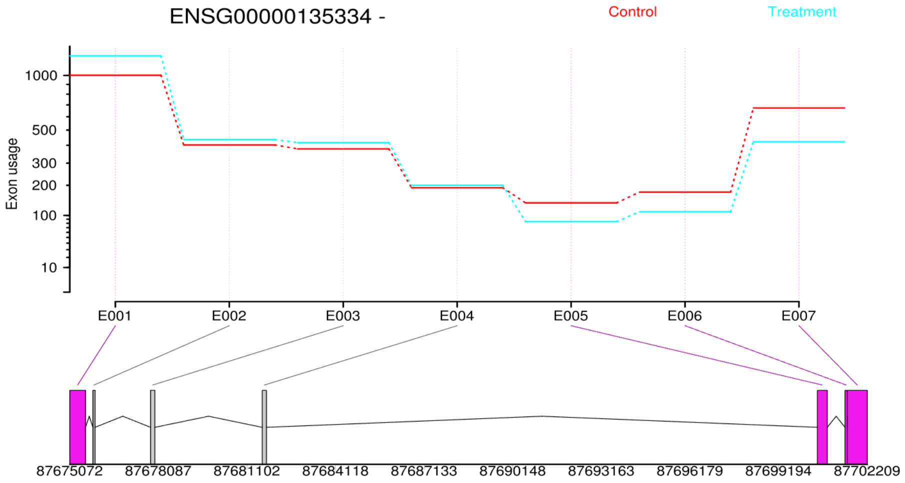

Gene: ACTB ENSG00000075624 http://www.ensembl.org/Homo_sapiens/Gene/Summary?g=ENSG00000075624;r=7:5526409-5563902

Actin beta

geneID	chromosome	start	end	total_exons	exon_changes
ENSG00000075624	7	5527151	5563784	35	27

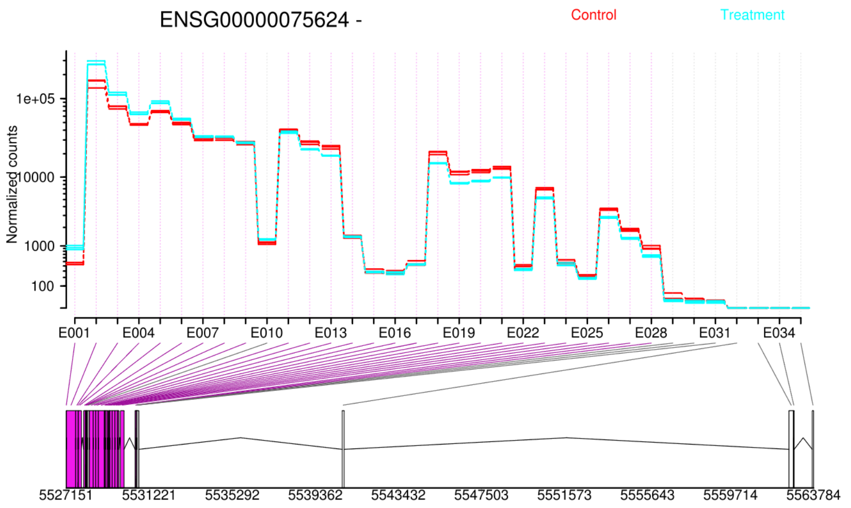

groupID	featureID	exonBaseMean	dispersion	pvalue	padj	seqnames	start	end	width	strand	Control	Treatment	log2fold_Treatment_Control
ENSG00000075624	E001	6.963013e+02	0.002	0.000	0.000	7	5527151	5527155	5	-	35.480	44.261	0.319
ENSG00000075624	E002	2.181485e+05	0.004	0.000	0.000	7	5527156	5527611	456	-	173.597	183.762	0.082
ENSG00000075624	E003	9.605891e+04	0.000	0.000	0.000	7	5527612	5527732	121	-	155.110	160.275	0.047
ENSG00000075624	E004	5.609611e+04	0.000	0.000	0.000	7	5527733	5527740	8	-	142.006	145.476	0.035
ENSG00000075624	E005	7.911048e+04	0.000	0.000	0.000	7	5527741	5527891	151	-	151.738	153.911	0.021
ENSG00000075624	E006	5.184762e+04	0.001	0.000	0.000	7	5528004	5528098	95	-	142.704	141.205	-0.015
ENSG00000075624	E007	3.193653e+04	0.001	0.000	0.000	7	5528099	5528110	12	-	130.763	127.949	-0.031
ENSG00000075624	E008	3.220047e+04	0.001	0.000	0.000	7	5528111	5528149	39	-	131.241	127.914	-0.037
ENSG00000075624	E009	2.752954e+04	0.001	0.000	0.000	7	5528150	5528185	36	-	127.767	123.271	-0.052
ENSG00000075624	E010	1.201880e+03	0.001	0.016	0.086	7	5528186	5528280	95	-	51.153	50.312	-0.024
ENSG00000075624	E011	3.885079e+04	0.001	0.000	0.000	7	5528281	5528469	189	-	137.481	131.454	-0.065
ENSG00000075624	E012	2.534351e+04	0.002	0.000	0.000	7	5528470	5528591	122	-	128.157	118.356	-0.115
ENSG00000075624	E013	2.163273e+04	0.002	0.000	0.000	7	5528592	5528719	128	-	124.672	113.509	-0.135
ENSG00000075624	E014	1.418387e+03	0.000	0.000	0.000	7	5528720	5529018	299	-	55.831	52.334	-0.093
ENSG00000075624	E015	2.896768e+02	0.005	0.005	0.036	7	5529019	5529059	41	-	29.120	26.349	-0.144
ENSG00000075624	E016	2.701858e+02	0.006	0.006	0.041	7	5529060	5529066	7	-	28.225	25.489	-0.147
ENSG00000075624	E017	4.455424e+02	0.004	0.000	0.004	7	5529067	5529160	94	-	35.505	31.794	-0.159
ENSG00000075624	E018	1.788959e+04	0.001	0.000	0.000	7	5529161	5529215	55	-	120.445	107.739	-0.161
ENSG00000075624	E019	9.854323e+03	0.001	0.000	0.000	7	5529216	5529234	19	-	105.203	92.637	-0.184
ENSG00000075624	E020	1.041629e+04	0.001	0.000	0.000	7	5529235	5529281	47	-	106.446	94.240	-0.176
ENSG00000075624	E021	1.151818e+04	0.001	0.000	0.000	7	5529282	5529400	119	-	108.888	96.913	-0.168
ENSG00000075624	E022	3.560511e+02	0.005	0.000	0.000	7	5529401	5529534	134	-	32.717	28.160	-0.216
ENSG00000075624	E023	6.117313e+03	0.001	0.000	0.000	7	5529535	5529663	129	-	92.857	81.437	-0.189
ENSG00000075624	E024	4.622449e+02	0.003	0.000	0.000	7	5529664	5529733	70	-	36.574	31.841	-0.200
ENSG00000075624	E025	2.018611e+02	0.004	0.000	0.000	7	5529806	5529982	177	-	25.309	21.624	-0.227
ENSG00000075624	E026	3.241848e+03	0.001	0.000	0.000	7	5530524	5530583	60	-	77.333	66.713	-0.213
ENSG00000075624	E027	1.593605e+03	0.001	0.000	0.000	7	5530584	5530590	7	-	61.458	51.306	-0.260
ENSG00000075624	E028	7.894736e+02	0.001	0.000	0.000	7	5530591	5530601	11	-	47.544	38.042	-0.322
ENSG00000075624	E029	1.853642e+01	0.084	0.019	0.101	7	5530602	5530602	1	-	8.874	5.486	-0.694
ENSG00000075624	E030	1.065375e+01	0.014	0.008	0.051	7	5530603	5530604	2	-	6.485	4.561	-0.508
ENSG00000075624	E031	9.043738e+00	0.012	0.041	0.170	7	5530605	5530709	105	-	5.865	4.457	-0.396
ENSG00000075624	E032	0.000000e+00				7	5540676	5540771	96	-			
ENSG00000075624	E033	0.000000e+00				7	5562574	5562790	217	-			
ENSG00000075624	E034	0.000000e+00				7	5562791	5562828	38	-			
ENSG00000075624	E035	0.000000e+00				7	5563714	5563784	71	-			

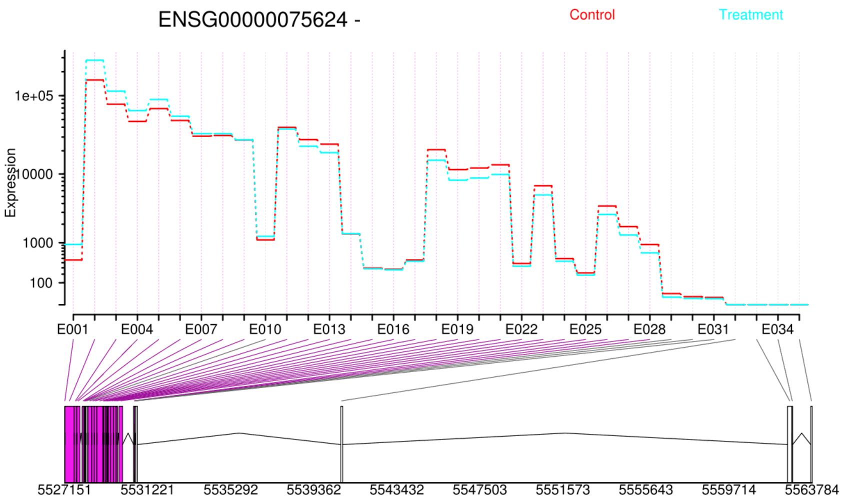

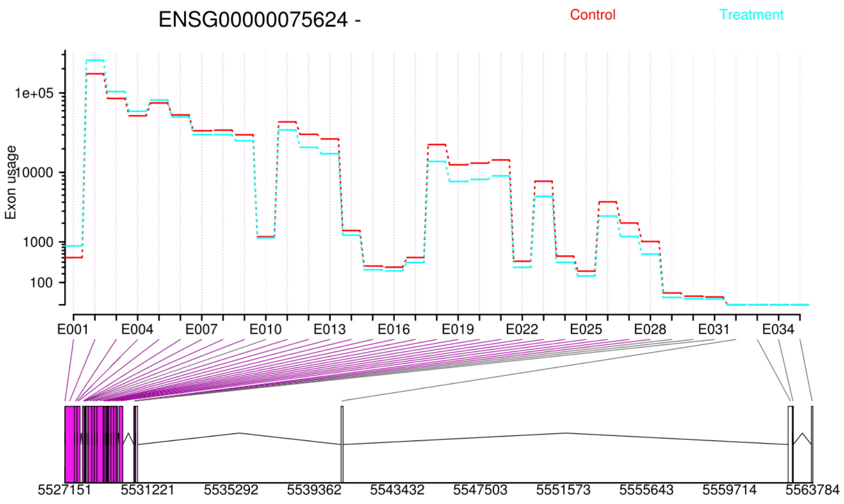

Geneid	Chr	Start	End	Strand	Length	WT3	WT2	WT1	KO2	KO3	KO1
"ENSG00000135334"	"6"	87675072	87675607	"-"	536	1347	1039	1430	1080	843	1136
"ENSG00000135334"	"6"	87675860	87675931	"-"	72	605	410	530	385	300	383
"ENSG00000135334"	"6"	87677818	87677967	"-"	150	529	426	495	367	306	346
"ENSG00000135334"	"6"	87681620	87681763	"-"	144	251	217	256	160	172	154
"ENSG00000135334"	"6"	87700510	87700846	"-"	337	212	121	196	62	59	86
"ENSG00000135334"	"6"	87701450	87701525	"-"	76	267	201	198	103	90	75
"ENSG00000135334"	"6"	87701526	87702209	"-"	684	1126	770	694	374	295	361
"ENSG00000075624"	"7"	5527151	5527155	"-"	5	651	392	523	924	749	913
"ENSG00000075624"	"7"	5527156	5527611	"-"	456	187441	153005	186862	270705	218653	284410
"ENSG00000075624"	"7"	5527612	5527732	"-"	121	102101	72995	88312	108854	89786	118393
"ENSG00000075624"	"7"	5527733	5527740	"-"	8	63836	43579	52825	61453	51136	67745
"ENSG00000075624"	"7"	5527741	5527891	"-"	151	92372	64553	75438	84766	70367	93738
"ENSG00000075624"	"7"	5528004	5528098	"-"	95	67391	45835	51478	51363	43362	58592
"ENSG00000075624"	"7"	5528099	5528110	"-"	12	44195	28113	32375	30806	26191	35167
"ENSG00000075624"	"7"	5528111	5528149	"-"	39	44842	28943	32748	30586	26297	35143
"ENSG00000075624"	"7"	5528150	5528185	"-"	36	39485	25149	28654	25707	21904	29331
"ENSG00000075624"	"7"	5528186	5528280	"-"	95	1557	1066	1168	1160	1020	1399
"ENSG00000075624"	"7"	5528281	5528469	"-"	189	56653	37038	41549	35325	29556	40699
"ENSG00000075624"	"7"	5528470	5528591	"-"	122	39969	25792	28865	20822	18208	24627
"ENSG00000075624"	"7"	5528592	5528719	"-"	128	35026	22490	25244	17270	15143	20294
"ENSG00000075624"	"7"	5528720	5529018	"-"	299	1866	1369	1531	1286	1124	1554
"ENSG00000075624"	"7"	5529019	5529059	"-"	41	463	257	292	248	233	313
"ENSG00000075624"	"7"	5529060	5529066	"-"	7	424	248	271	220	218	302
"ENSG00000075624"	"7"	5529067	5529160	"-"	94	715	407	456	396	356	445
"ENSG00000075624"	"7"	5529161	5529215	"-"	55	28996	19578	21473	13598	12192	16276
"ENSG00000075624"	"7"	5529216	5529234	"-"	19	16027	10837	11798	7412	6759	8926
"ENSG00000075624"	"7"	5529235	5529281	"-"	47	16593	11410	12544	7909	7150	9591
"ENSG00000075624"	"7"	5529282	5529400	"-"	119	18078	12566	13943	8884	7930	10611
"ENSG00000075624"	"7"	5529401	5529534	"-"	134	584	354	369	285	279	352
"ENSG00000075624"	"7"	5529535	5529663	"-"	129	9963	6334	7452	4923	4168	5524
"ENSG00000075624"	"7"	5529664	5529733	"-"	70	749	437	500	426	330	447
"ENSG00000075624"	"7"	5529806	5529982	"-"	177	328	179	237	160	159	201
"ENSG00000075624"	"7"	5530524	5530583	"-"	60	5218	3394	3917	2625	2212	2940
"ENSG00000075624"	"7"	5530584	5530590	"-"	7	2645	1686	1917	1258	1054	1453
"ENSG00000075624"	"7"	5530591	5530601	"-"	11	1243	926	966	585	501	723
"ENSG00000075624"	"7"	5530602	5530602	"-"	1	18	42	20	8	9	15
"ENSG00000075624"	"7"	5530603	5530604	"-"	2	17	17	11	7	4	11
"ENSG00000075624"	"7"	5530605	5530709	"-"	105	17	10	10	6	4	11
"ENSG00000075624"	"7"	5540676	5540771	"-"	96	0	0	0	0	0	0
"ENSG00000075624"	"7"	5562574	5562790	"-"	217	0	0	0	0	0	0
"ENSG00000075624"	"7"	5562791	5562828	"-"	38	0	0	0	0	0	0
"ENSG00000075624"	"7"	5563714	5563784	"-"	71	0	0	0	0	0	0
